# A First-Principles Study of l‑Glutathione Adsorption on Chiral Gold Nanoparticles

**DOI:** 10.1021/acsmaterialsau.5c00262

**Published:** 2026-05-25

**Authors:** Jianming Cui, Xin Qi

**Affiliations:** Department of Chemistry, 3728 Dartmouth College, Hanover, New Hampshire 03755, United States

**Keywords:** Au nanoparticle, chiral nanoparticle, density functional theory, molecular adsorption, binding energy, enantioselectivity

## Abstract

The chiral 432 helicoids II and III gold (Au) nanoparticles have been successfully synthesized using l-glutathione (L-GSH) as the chiral shape modifier; however, how L-GSH interacts with Au surfaces to trigger and promote the formation of chiral shapes remains unknown. In this work, the selectivity of L-GSH on enantiomeric Au(321)^*R*/*S*^ is investigated using first-principles density functional theory (DFT). To avoid a resource- and time-consuming brute-force geometric scan of a full L-GSH molecule adsorbed on Au(321)^*R*/*S*^, we first decomposed L-GSH into three amino acid analogues, namely, l-glutamic acid (l-Glu), l-cysteine (l-Cys), and glycine (Gly), and reduced the chiral Au(321)^*R*/*S*^ facets into constituent microfacets, namely, Au(111), Au(100), and Au(110). By understanding how each molecular building block interacts with these three low-Miller-index facets, we rationally designed six sets of initial configurations of L-GSH adsorbed on Au(321)^*R*/*S*^ and performed optimization to identify the most thermodynamically stable structures. We find that L-GSH enantioselectively binds more strongly to Au(321)^*R*^, but the enantiomeric shift in binding energy is small. Regardless of the facet chirality, all strong-binding configurations exhibit a “reach-and-stretch” mechanism to maximize contact between L-GSH and kinks, multiple steps, and terraces, where both thiol and amine groups play critical roles. The enantioselectivity reported herein supports experiments where L-GSH is observed to promote the expression of Au(321)^*R*^, and the laterally extended conformation of L-GSH on Au(321) can inspire future studies on the roles of L-GSH in inducing chiral shape formation.

## Graphical abstract

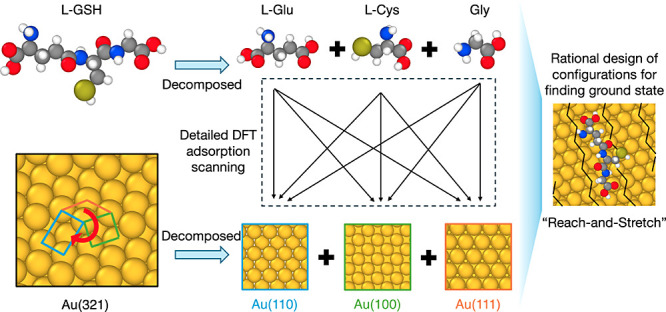

## Introduction

Chiral shapes can induce changes in electron spin polarization, thereby generating circularly polarized light. For nanometer-sized chiral structures composed of plasmonic materials, such as noble metals, this effect can be significantly amplified due to the near-field enhancement imparted from the intrinsic localized surface plasmon resonance (LSPR) associated with metal nanocrystals.^1^ Over the past decade, the emerging field of chiral plasmonic nanomaterials has attracted growing attention for their promising applications in sensing, photocatalysis, photoelectronics, and photodynamic therapy.^2–6^ For instance, the substantially different immune responses observed in vivo and in vitro to chiral versus achiral metal nanoparticle sensors drive the more precise engineering of chiral inorganic nanostructures to better understand biological systems.^7^ Chiral plasmonic nanostructures with high dissymmetry factors (g-factors) have shown remarkable efficiency in converting solar energy into clean fuel through photocatalysis.^8^ Additionally, a photodynamic therapy utilizing a chiral photosensitizer that generates singlet oxygen (^1^O_2_) has been reported to perform at least three times better than standard organic photosensitizers.^6^

While chirality is ubiquitous in biological systems, metals, especially when arranged atomically in face-centered cubic (fcc) lattices, lack chiral centers capable of forming chiral structures. In early attempts, the synthesis of chiral plasmonic nanoparticles was typically achieved through assembly, in which achiral plasmonic nanoparticles were assembled into chiral configurations guided by chiral molecular templates, such as DNA and proteins.^9–12^ Similarly, the metal–molecule–metal (3M) approach produces chiral products with chiral molecules located in the nanogaps between the inner metal core and the outer metal shells, where the gold (Au) nanoparticles with strongly binding chiral biomolecules are predominantly used.^13–15^ The synthesis of individual chiral nanoparticles has been realized more recently: González-Rubi and co-workers synthesized Au nanorods with highly chiral-optically active wrinkles via micelle-directed growth.^16^

Inspired by the seed-mediated growth of noble metal nanocrystals, which offers a spatiotemporal separation of nucleation and growth, an aqueous-based, two-step growth method was reported to synthesize enantiomerically pure chiral Au nanoparticles using enantiomerically pure amino acids or short peptides by harnessing the enantiospecific interactions between organic ligands and high-Miller-index facets.^17–20^ The process where chiral ligands assist the growth of achiral seeds, such as cubes and octahedra, into chiral nanoparticle products is known as chirality transfer, and these ligands are referred to as the chiral shape modifiers. After introducing cysteine (Cys) or glutathione (GSH), 432 helicoid Au nanoparticles are formed, with shapes specific to the seed and chiral ligand pairs. For example, 432 helicoid Au II is formed by adding GSH to cubic Au seeds, and 432 helicoid Au III, with a high g-factor up to approximately 0.2 at 622 nm, is formed by adding GSH to octahedral Au seeds.^17^ The handedness of the chiral ligand deterministically governs the handedness of the Au nanoparticle, which is manifested as a dominant expression of one of the enantiomeric forms of the chiral facets.

The concept of chiral facets was first introduced by Gellman et al. in 1996,^21^ which defines high-Miller-index fcc facets with unequal step lengths on each side of the kinks as chiral. A naming system was proposed in analogy to the Cahn–Ingold–Prelog rules, where the priority is given to the long step > the short step > the terrace below the step. The surface is labeled as *R* (*S*) if the circle centered at the kink site is drawn in a clockwise (counterclockwise) orientation. Attard further extended the definition to include all possible chiral surfaces, exemplified by Pt(531), with equal lengths on either side of the kink sites.^22^ A revised notation was proposed: when viewing the kink site from above, the surface is denoted as *R* (*S*) if the sequence (111) → (100) → (110) runs clockwise (counterclockwise) (see Figure 1a). Sholl noted that Gellman’s and Attard’s notations are not always compatible, and Attard’s notation is more favored because Gellman’s notation cannot describe chiral surfaces with equal lengths on each side of the kink sites, such as fcc(531).^23^

**Figure 1:**
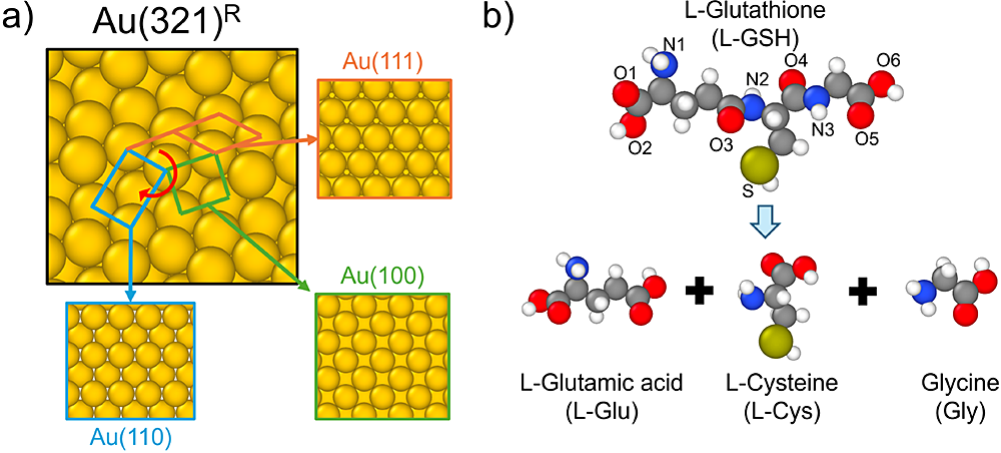
(a) Example of the Au(321)^*R*^ facet composed of Au(111) marked in a pink outline, Au(100) marked in a green outline, and Au(110) marked in a blue outline. The red arrow represents Attard’s notation for handedness, which is *R* in this example.^22^ (b) L-GSH molecule decomposed into l-Glu, l-Cys, and Gly. The C atoms are shown in gray, O atoms in red, N atoms in blue, S atoms in lime, and H atoms in white. The numbering of all S, N, and O atoms is used for later analysis.

In general, any high-Miller-index facet {*hkl*} can be decomposed into three independent vectors,^24^ as shown in

$$\{hkl\}=a_{1}\vec{u_{1}}+a_{2}\vec{u_{2}}+a_{3}\vec{u_{3}}$$ 1

where $\vec{u_{1}}$ is fixed to be {111}, $\vec{u_{2}}$ to be {110}, and $\vec{u_{3}}$ to be {100}.^23^ For example, fcc(643) can be decomposed into 3{111} + 1{110} + 2{100}. This decomposition of a high-Miller-index facet into microfacets with low Miller indices can uniquely identify a chiral facet: any fcc­(*hkl*) surface with *h* ≠ 0, *k* ≠ 0, *l* ≠ 0, and *h* ≠ *k* ≠ *l* is chiral.^23,24^ The ratio *n*_*i*_ of the low-Miller-index microfacets can be written as

$$n_{111}:n_{110}:n_{100}=4a_{111}:2a_{110}:2a_{100}$$ 2

where *a*_*i*_ is calculated from eq 1. The microfacet with the largest *n* forms the terrace, and the other two facets constitute the kinked steps. For example, given *a*_111_:*a*_110_:*a*_100_ = 3:1:2 for fcc(643), it consequently follows *n*_111_:*n*_110_:*n*_100_ = 6:1:2; thus, one fcc(643) unit cell is made up of six unit cells of fcc(111) as a terrace, one unit cell of fcc(110) and two unit cells of fcc(100) as a kinked step.

When 432 helicoids I and II grow from cubic seeds with l-cysteine (l-Cys) and l-glutathione (L-GSH), respectively, it was found that both shape evolutions involve a shift from the *R* region (predominantly Au(321)^*R*^) to the *S* region (predominantly Au(321)^*S*^), but at different *R* – *S* boundaries.^17^ They postulate that this is likely due to differences in molecular sizes and the flexibility provided by the γ-peptide linkage, such that the amine and thiol groups on l-Cys interact with a single kink, whereas those on L-GSH interact with multiple kinks.^17^ They further hypothesize that it is the strong, selective interaction between l-Cys and the Au(321)^*R*^ that inhibits the vertical growth and promotes the lateral growth, thus shifting the *R* – *S* boundaries.^19^ However, due to limitations in temporal and spatial resolution, how chiral ligands interact with chiral facets and impart chiral shape development remains difficult to probe with purely experimental techniques.

Numerous theoretical studies using first-principles density functional theory (DFT) have examined the interactions between L- and d-Cys and various chiral facets, shedding light on chiral recognition and enantioselectivity. In these studies, the adsorption of the Cys radical or thiolated Cys on flat or chiral surfaces was mostly investigated.^25–28^ They found the optimized positions of the S atom to be the short-bridge site on Au(110),^25^ the bridge site on Au(111) and Au(321)^*R*/*S*^,^26^ the off-top site on Au(111),^27^ and the kinked site on Au(17 11 9)^*S*^.^28^ They showed that the relative position of the amine to the thiol group could determine the binding preferences to the *R* or *S* kinked sites.^28,29^ Fajín and co-workers reported that l-Cys binds to Au(321)^*R*^ 0.08 eV more strongly than to Au(321)^*S*^ when the generalized gradient approximation (GGA) by Perdew, Burke, and Ernzerhof (PBE) with dispersion correction D2 (i.e., PBE-D2) was used.^26^ Their work also demonstrates the importance of considering dispersion in DFT calculations, as the trend was reversed if the PW91 potential alone was used.

In more recent work,^30^ a comprehensive analysis was conducted on various forms of Cys, including neutral Cys without losing the proton, thiolated Cys, conventional zwitterionic Cys by transferring the proton from the carboxylic group to the amine with thiol and thiolate groups, and unconventional zwitterionic Cys molecules where the proton is transferred from the thiol group to the N atom. On a flat Au(111) surface (coordination number (CN) = 9), the unconventional zwitterionic structure is found to be the most stable with the S atom at the off-top site, while the neutral molecule with an undissociated thiol group is the second most stable, with the S atom at the atop site. The adsorption configuration of thiolated Cys is optimized with the S atom at the bridge site, which is consistent with earlier studies.

Despite the abundant theoretical studies on Cys, DFT studies that report the selectivity of GSH toward enantiomeric chiral facets are scarce, if not nonexistent. This is mainly due to the prohibitively expensive computation for a brute-force geometric scan of a large, flexible molecule on a complex surface. In this work, we look into the L-GSH-facilitated formation of 432 helicoid II and III Au nanoparticles, which express Au(321)^*R*/*S*^ in high-Miller-index facet regions.^17^ As the initial step to understand the roles of chiral shape modifiers, we use dispersion-corrected DFT to investigate how L-GSH interacts with Au(321)^*R*^ and Au(321)^*S*^ facets and whether an enantioselective adsorption exists. Instead of a brute-force geometric scan, we first study how each amino acid building block on a full L-GSH interacts with one of the three low-Miller-index microfacets and rationally build a list of candidate configurations of L-GSH adsorbed on Au(321)^*R*/*S*^ for further optimization. We find that the enatio-adsorption preference of L-GSH is slightly stronger on Au(321)^*R*^ than on Au(321)^*S*^. Interestingly, only interacting with multiple kink sites does not guarantee a strong interaction. In fact, the thiol and amine groups need to interact with kinks at different levels of terraces to increase the binding affinity, as such an extended conformation maximizes contact between heteroatoms and the {111} terrace and lowers the binding energy.

## Computational Methods

### System Setup

To avoid a highly resource- and time-consuming search of ground-state configurations for a full L-GSH molecule adsorbed on high-Miller-index facets, we first reduced the dimensionality of Au(321)^*R*/*S*^ and L-GSH to investigate the interaction among the building blocks. For Au, we focused on Au(100), Au(111), and Au(110) because according to eq 2, one unit cell of Au(321) is composed of one unit cell of Au(110) and one unit cell of Au(100) as steps and two unit cells of Au(111) as a terrace (see Figure 1a). Similarly, we dissected the tripeptide L-GSH into three building blocks and represented each using the closest amino acid analogue, namely, l-Glu, l-Cys, and glycine (Gly) (Figure 1b). Considering the proton-rich synthetic environment (e.g., pH≈3) and the recent work by Idris and co-workers on the various forms of Cys,^17,20,30,31^ we consider a thiol group, instead of a thiolate group, in our GSH setup.

Following the dimensionality reduction, the adsorption behaviors of each amino acid on Au(111), Au(100), and Au(110) were investigated, including configurations, bond lengths, binding energies, site preferences, and bond types. The search for optimized adsorption configurations was performed by rotating and translating the molecule on Au facets, and over 220 DFT calculations were performed for amino acids on low-Miller-index facets. The scanning procedure proceeded as follows. We first performed several trial runs to identify the most important heteroatoms on each amino acid, i.e., S for l-Cys, N for Gly/l-Glu. Next, we determined the likely preferred orientation of the molecule relative to the surface, e.g., standing vs lying, while placing the most important heteroatoms on their preferred site found in the first step. Then, based on the likely orientation, we rotated the molecule within the plane parallel to the surface with an increment of 15°. The scanned angles range from 0 to 60° for Au(111), 90° for Au(100), and 180° for Au(110). Finally, the optimized configuration from rotation was translated on the Au surface by shifting the most important heteroatoms toward different sites, including the atop, bridge, fcc, and hexagonal close-packed (hcp) sites for Au(111), atop, bridge, and hollow sites for Au(100), and atop, short-bridge, and long-bridge sites, as well as vertically into the troughs, for Au(110).

After identifying the site preference and bond type between the amino acid and low-Miller-index facet for each pair, we rationally designed the initial configurations of L-GSH on Au(321)^*R*/*S*^ for the optimization. Such a workflow provides an efficient and cost-effective way to search for preferred adsorption configurations of a large molecule on a complex metal surface.

### First-Principles DFT Calculations

All DFT calculations were conducted using the Vienna Ab initio Simulation Package (VASP).^32,33^ The generalized gradient approximation by Perdew, Burke, and Ernzerhof (PBE) was used for the exchange-correlation function.^34^ Damping parameters and projector augmented wave (PAW) pseudopotentials were used.^35,36^ The energy cutoff for the plane-wave basis was set to be 450 eV, and the global break condition of the electronic SC-loop was 1 × 10^–6^ eV. Monkhorst–Pack grids were applied for the sampling of the first Brillouin zone.^37^ The DFT-D3 method^38^ with Becke–Johnson damping^39^ was used to describe long-range van der Waals interactions.

The binding energies of molecules adsorbed on Au surfaces are calculated using the following equation:

$$E_{B}=E_{\mathrm{ads}}-(E_{\mathrm{mol}}+E_{\mathrm{slab}})$$ 3

where *E*_B_ is the binding energy, *E*_ads_ is the energy of an optimized configuration of a molecule adsorbed on a Au facet, *E*_mol_ is the energy of a single molecule in vacuum, and *E*_slab_ is the energy of a relaxed slab of the corresponding Au facet.

The value of *E*_mol_ was calculated by placing a single molecule in a cubic unit cell with a length of 30 Å with a k-point of 1 × 1 × 1. To calculate *E*_slab_, we first determined the lattice constant for Au with the above functionals by optimizing a 2 × 2 × 2 bulk Au with a k-point mesh of 6 × 6 × 6. The lattice constant was found to be 4.10 Å, which is in good agreement with the experimental value of ∼4.07 Å.^40–42^ Based on the obtained lattice constant, semiperiodic Au slabs of {100}, {111}, {110}, and {321} facets with a 30 Å vacuum above were prepared and relaxed. The top 4 layers of the optimized slabs are taken and fixed for further binding energy calculation. A k-point of 4 × 4 × 1 was used for systems with semiperiodic slabs. See the Supporting Information (SI) for more details.

To obtain the total density of states (DOS) and projected density of states (PDOS) of the interested molecules and the most important heteroatoms in vacuum and on a Au surface, calculations were continued from the optimized configurations with an increased k-point of 8 × 8 × 1 for more precise DOS data. The PDOS captures electron redistribution in the molecule upon adsorption due to its interaction with the metal *d* orbital. The change in the local electronic structure is manifested as shifted and/or broadened peaks, as well as the emergence of antibonding states above the Fermi level in strong covalent bonding.^43^

## Results and Discussion

### Amino Acid Analogue Adsorption on Low-Miller-Index Microfacets

To understand how each amino acid building block on an L-GSH molecule interacts with the composing unit of a chiral Au facet, we carefully examine the conformations as well as electronic properties of the amino acid analogues in their most thermodynamically stable state on the three microfacets. The relative positions of heteroatoms to the surface layer Au atoms later serve as the basis for designing the likely preferred adsorption configurations of L-GSH on Au(321)^*R*/*S*^. Below, we elaborate on the key findings.

#### Adsorption Configurations and Thermodynamics

To compare the adsorption behaviors of amino acid analogues across various facets, we selected the optimized configuration with the lowest *E*_B_ for each amino acid and microfacet pair and summarized them in Figure 2. In addition to these ground states, we report three additional adsorption configurations before and after geometry optimization for each pair in Figures S2–S10 in the Supporting Information to assist in illustrating general trends of preferred binding sites and the contribution of different heteroatoms in binding.

**Figure 2:**
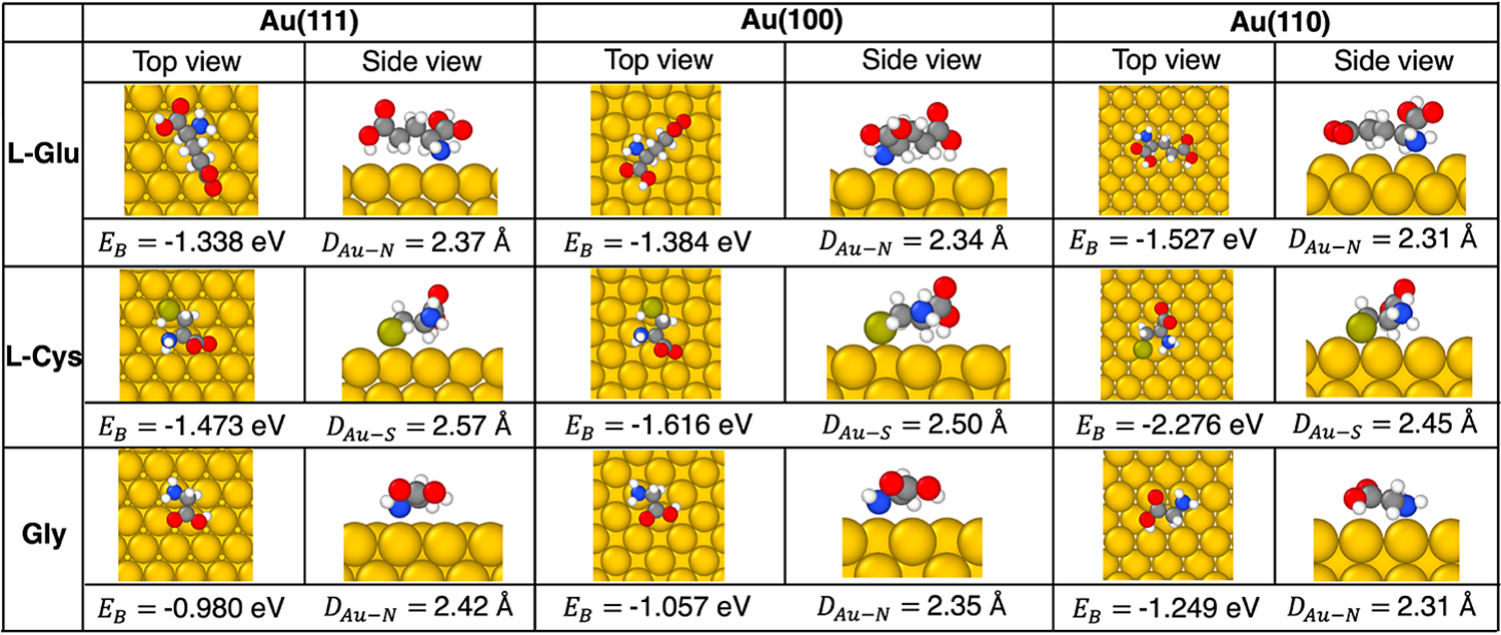
Summary of each amino acid and low-Miller-index facet pair with the lowest binding energy *E*_B_ selected from the ground-state search. For each of the most thermodynamically stable states, we show the adsorption configurations in the top and side views, along with the binding energy *E*_B_ and the bond length between the most important heteroatom and Au, *D*_Au–x_. The C atoms are shown in gray, O atoms in red, N atoms in blue, S atoms in lime, H atoms in white, and Au atoms in gold.

During geometry optimization, we note that not all heteroatoms in the amino acids move toward the surface. Rather, only the N atom on l-Glu, the S atom on l-Cys, and the N atom on Gly exhibit a tendency to minimize their distance to a surface Au atom, identifying themselves as the most important heteroatom of each amino acid (see the side views in Figures 2 and S2–S10). The bond length between these most important heteroatoms and the closest Au surface atom is listed for each configuration in Figure 2. In contrast, none of the O atoms shows more favored binding compared to the S and N atoms, as they are usually located higher from the surface and may move away from the surface during optimization (see Figures S3 and S9). For Au(100) and Au(111) facets, the most important heteroatoms in all three amino acid analogues preferably adopt the atop (N atoms on l-Glu and Gly) or off-top (S atom on l-Cys) site, as suggested by the top views in Figure 2. Such a preference persists for l-Glu and Gly on Au(110) as both N atoms are still located on top of a ridge atom; however, the S atom on the l-Cys prefers a short-bridge site on Au(110). In addition, we find that, at the end of geometry optimization, the −SH thiol group remains for l-Cys adsorbed on Au(100) and Au(111), but chemically transformed to −S^–^ thiolate for that on Au(110), with the proton being transferred to the nearby N atom. The conversion from the neutral molecule to the unconventional zwitterionic molecule not only alters the preferred binding site of the S atom on Au, but also significantly increases the binding energy to −2.276 eV, making it the most thermodynamically stable pair among the nine in Figure 2.

In previous studies, the adsorption of Cys on flat and stepped Au surfaces has typically been carried out in a thiolated form, in which the S atom was found to preferentially occupy bridge sites on both Au(110) and Au(111).^26–28,44^ We keep the neutral l-Cys form with the thiol group in the initial configurations in this work, considering the proton-rich environment in the synthesis of 432 helicoid II and III at pH ∼ 3.^17,20,31^ Further, a recent study comparing the binding of several Cys forms on clean and nanostructured Au(111) facets, including the neutral thiol and thiolate forms, the so-called unconventional zwitterion (same as the structure we found in the Au(110)) form, and two additional zwitterions of Cys, has found that the neutral thiol form binds more strongly to clean Au(111), while thiolate generally prefers to less-coordinated sites.^30^ Their finding on the preferred off-top site for neutral thiol is also consistent with our results.

Next, we assess the likely contribution of each amino acid building block to the overall binding of L-GSH based on Au surfaces. By comparing *E*_B_ of different amino acid analogues in the same column shown in Figure 2, we find a general trend of l-Cys > l-Glu > Gly for all facets. This result is expected, considering that for amino acids with the same most important heteroatoms (e.g., N), l-Glu contains more electron-rich atoms (e.g., O) that may serve as potential electron donors to less-coordinated surface Au atoms and that S, particularly in thiol and thiolate, is well-known for its ability to form covalent bonds with Au atoms.^25,27,30^

For amino acids with N as the most important heteroatom, the binding energy *E*_B_ of l-Glu is consistently 0.3 eV stronger than that of Gly across all facets, with the difference on Au(110) being the smallest (i.e., Δ*E*_B,Glu–Gly,{111}_ = 0.358 eV, Δ*E*_B,Glu–Gly,{100}_ = 0.327 eV, and Δ*E*_B,Glu–Gly,{110}_ = 0.278 eV). The binding energy difference between l-Cys and the second preferred l-Glu increases with the facet and most pronounced for Au(110) (i.e., Δ*E*_B,Cys–Glu,{111}_ = 0.135 eV, Δ*E*_B,Cys–Glu,{100}_ = 0.232 eV, and Δ*E*_B,Cys–Glu,{110}_ = 0.749 eV). The small increase from Au(111) to Au(100) is likely due to shortened bond length (i.e., Δ*D*_Au–N,Glu,{100}–{111}_ = −0.03 Å vs Δ*D*_Au–S,{100}–{111}_ = −0.07 Å), the trend of which has been similarly observed between l-Glu and Gly (i.e., Δ*D*_Au–N,Gly,{100}–{111}_ = −0.07 Å). The much stronger binding of l-Cys on Au(110) can be attributed to the change of a neutral molecule to an unconventional zwitterionic molecule, as similarly reported in Idris et al.^30^ Further discussion is provided in later sections.

In addition to informing the relative contribution of the amino acid building blocks to the overall binding of L-GSH on Au facets, Figure 2 also reveals surface sensitivity of binding, providing critical information for building the initial configuration to optimize the geometry of a full L-GSH on chiral Au facets, as a part of the main rationale for this study. Again, all amino acids exhibit the same facet selectivity, such that the magnitude of bining strengths follows *E*_B,{110}_ > *E*_B,{100}_ > *E*_B,{111}_. Given that all chiral surfaces are a combined expression of these three facets, and Au(321) has Au(100) and Au(110) as the steps and Au(111) as the terrace, we will prioritize the discussed S and N atoms near the kink or close to the step edge, instead of remaining on the terrace, in L-GSH-Au­(321) initial configuration construction. Furthermore, we postulate that a preferred binding on Au(100) rather than on Au(111) for all amino acids is a likely contributing factor to promoting the formation of the cubic outline on 432 helicoid III via thermodynamic control.

#### Bond Types of Key Atoms in Amino Acids with Au Surfaces

A scrutiny of the adsorbed configurations on the low-Miller-index microfacets reveals the most critical atom responsible for adsorption for each amino acid analogue. To understand how the interaction between the N or S atom and the Au surface leads to different degrees of preferred binding, we turn to the DOS and PDOS, charge density difference, and charge-transfer analyses to provide crucial information about the electronic structure of the system, such as electron redistribution and bond types.^25,30,45^

Figure 3 includes the total DOS of free molecules for all amino acids, their PDOS when adsorbed on the indicated surface, and, more importantly, the PDOS projected onto the most important heteroatom identified above (i.e., S for l-Cys and N for l-Glu and Gly). The energy range is shown from −10 to 2 eV, with the Fermi level set to zero. This range is chosen based on the energy levels of the Au *d* orbital, about 2–6 eV below the Fermi level, as suggested by the DOS of a bare Au surface in Figure S1b and reported by previous studies.^25,46^ The DOS of a bare Au(321) surface and the PDOS of less important heteroatoms (O atoms in Glu and Gly, N and O atoms in Cys) are included in Figure S11 in the Supporting Information.

**Figure 3:**
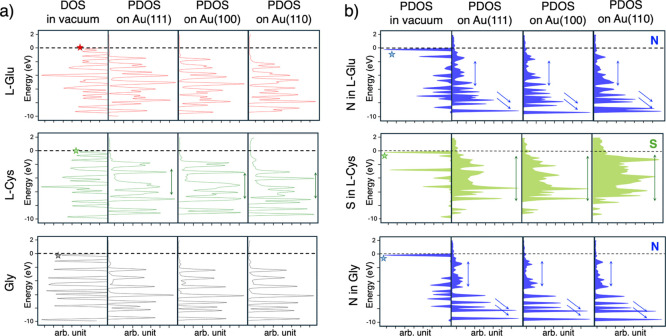
(a) Leftmost panel shows the DOS of isolated l-Glu (red), l-Cys (green), and Gly (black), and the right three panels show the PDOS of molecules in the adsorbed configuration listed in Figure 2. (b) PDOS of the most important heteroatoms l-Glu, l-Cys, and Gly in the isolated state (the leftmost panel) and in adsorbed configurations (the right three panels). The Fermi level is set to zero, marked with black dashed lines. The colored stars represent the HOMO peak in an isolated molecule. The single-head arrows suggest peak shifting, and the double-head arrows suggest the broadening areas of energy states.

When a strongly interacting ligand adsorbs onto a favored surface, an electron redistribution often occurs in the adsorbate. This is typically observed in the PDOS of the adsorbed structure, where the highest occupied molecular orbital (HOMO) disappears or weakens (indicating hybridization), peaks shift (indicating electron transfer), and peaks both shift and broaden (indicating chemisorption) compared to its isolated gas-phase state. Furthermore, an even stronger bonding is indicated if the antibonding states shift up through the Fermi level and become empty.^43^ The total DOS of the three amino acids in their isolated states is shown in the leftmost panels of Figure 3a,b. In comparison with the three adsorbed states on the right, all amino acids show a disappearance of the sharp HOMO peak on all three facets, as marked with colored stars but with varying degrees of peak shifting and broadening, suggesting different types of bonds formed between the adsorbate and the surface upon adsorption.

Among the three amino acids, Gly exhibits mostly entirely peak shifting and slight broadening in the range of −2 to −5 eV, overlapping with the energy levels of the Au *d* orbital. Similarly, l-Glu is characterized primarily by peak shifts, especially on Au(111) and Au(100). The peak broadening is slightly more pronounced when it is adsorbed on Au(110). The trend observed in the l-Glu PDOS is consistent with the trend in its binding energy reported in Figure 2, where *E*_B_ is comparable for Au(100) and Au(111) but is significantly higher for Au(110). In contrast, the change of PDOS for l-Cys is predominantly characterized by peak broadening between −2 and −8 eV, with less obvious peak shifting as a result, which indicates that l-Cys forms the strongest bonds with Au surfaces among these three amino acids. This finding is consistent with the results shown in Figure 2. The distinctive trends in the energy-level movement for amino acids with N and S being the most important atoms, respectively, have further motivated a more focused analysis of the PDOS projected onto the most important heteroatoms.

From Figure 3b, we again observe a disappearance of the HOMO peak in S for l-Cys and N for l-Glu and Gly on an isolated molecule upon adsorption. Since such a change is less pronounced or absent for other heteroatoms (see Figure S11 in the Supporting Information), this again confirms the important roles of the indicated atoms in hybridizing with the Au orbitals. By projecting the density of states only onto the S atom for l-Cys, it is clear that S strongly couples to the Au surface via chemisorption, both as a thiol or a thiolate, as the peaks below and above the Fermi level are substantially broadened. Such broadening is comparable for Au(111) and Au(100) but much more significant for Au(110), as the thiol–Au bond becomes a thiolate–Au bond. On the other hand, the N-projected DOS for both l-Glu and Gly on all facets shows clear downward shifts in all energy states as well as a consistent minor broadening between 0 and −5 eV, suggesting minor electron transfer and direct bonding. Notably, peak broadening has been observed for the PDOS of l-Glu and Gly, as well as the PDOS of their N atoms, but not for the O atoms therein (Figure S11).

Overall, the bonding between l-Cys and all low-Miller-index Au facets is mainly localized at the S atom, as indicated by a broadened HOMO rearrangement at Au *d* orbitals from PDOS plots. For l-Glu and Gly adsorption on Au surfaces, the bonding between the molecules and Au surfaces is mainly through N–Au interactions from the N atom, e.g., peak shifting in PDOS, and considerable HOMO broadening. Our findings are consistent with the bonding behaviors of the Cys radical and Gly on Au(110) reported by Bechstedt^25^ and the Cys adsorption on Au(111) reported by Paci.^30^

#### Charge Density Difference and Charge Transfer upon Adsorption

The charge density difference between the isolated and adsorbed states is investigated to further understand the bonding properties identified in the PDOS analysis. Its value, Δρ, can be calculated as

$$\Delta \rho =\rho_{\mathrm{ads}}-(\rho_{\mathrm{slab}}+\rho_{\mathrm{mol}})$$ 4

where ρ_ads_ is the charge density of the adsorbed system, ρ_slab_ is the charge density of a clean Au surface, and ρ_mol_ is the charge density of an isolated molecule that remains in the adsorbed configuration. That means the calculations for isolated molecules and slabs are performed on the same configurations as in the adsorbed state, rather than by optimization. The results for l-Cys on Au(110) and Gly on Au(111) are shown in Figure 4 for comparison.

**Figure 4:**
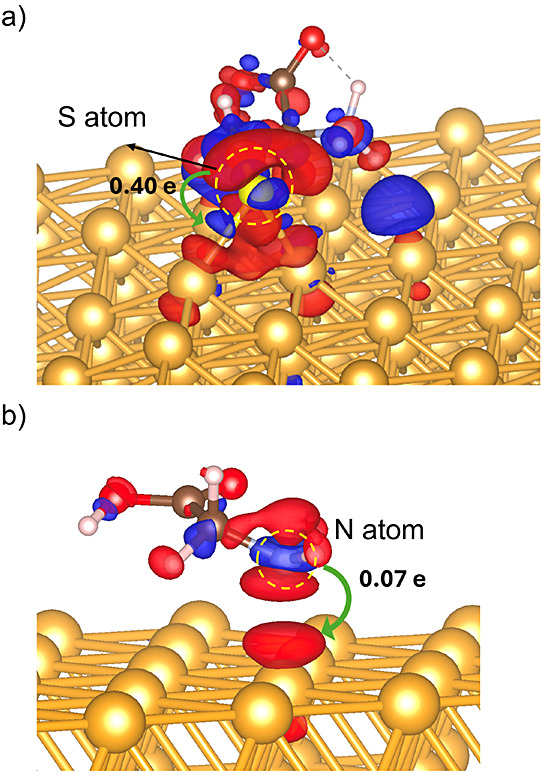
Charge density difference plots of (a) l-Cys adsorbed on Au(110) and (b) Gly adsorbed on Au(111). The blue and red regions represent the electron accumulation and depletion, respectively. Isosurface value: ±0.02 e/Å^3^.

The Bader charge-transfer calculations^47,48^ were performed for the two examples to further understand the changes in the charging state for the S and N atoms, using the Bader Charge Analysis by Henkelman.^49–52^ The VASP transition state theory tools (VTST Tools)^53^ were used to sum the core charge data and the valence charge data from VASP. The difference in the valence electronic charge of the target atom between the adsorbed molecule and the isolated molecule in the adsorbed configuration is calculated as

$$\Delta Q_{\mathrm{Bader}}=Q_{v,\mathrm{ads}}-Q_{v,\mathrm{mol}}$$ 5

where Δ*Q*_Bader_ is the difference in the Bader charge of the target atom, *Q*_v,ads_ is the valence charge of the target atom in the adsorbed molecule, and *Q*_v,mol_ is the valence charge of the target atom in the isolated form. The results are shown in Figure 4.

According to the periodic table from Sargent–Welch, the covalent radius of S and Au is 1.02 and 1.34 Å, respectively, leading to an estimated covalent S–Au bond length of 2.36 Å.^54^ This value is close to the S–Au bond lengths in our study, ranging from 2.45 to 2.57 Å, as shown in Figure 2. Figure 4a shows the charge density difference plot of the l-Cys adsorbed on Au(110) with the bond length of 2.45 Å and the electron transfer is found to be 0.40 e. The electron accumulation (blue) regions around the S atom form a tetrahedron shape, indicating the *sp* ^3^ orbital, and the electron depletion (red) regions of the surface Au atoms have the shape of the *d*_*z* ^2^_ orbital. Therefore, the thiolate S–Au bonding in l-Cys adsorption is a strong *sp* ^3^-*d*σ-type bond.

Similarly, the covalent bond length of N–Au is estimated to be around 2.09 Å by summing the covalent radii of N (0.75 Å) and Au (1.34 Å), which is close to the N–Au bond length range of 2.31 to 2.42 Å reported in Figure 2. However, different from l-Cys in Figure 4a, the charge density difference of Gly on Au(111) in Figure 4b does not indicate either covalent or ionic bonding between the amine group and Au surface. Although the electron redistribution centers around the amino group and the electron depletion on the surface Au also forms the shape of *d*_*z* ^2^_, there is only an electron depletion region between the N atom and the Au surface, which is different from the distribution of both electron accumulation and depletion in the thiolate S–Au bond. The electron transfer from the N atom is only 0.07 e, which is much less than that from the S atom to the S–Au bond, suggesting a much weaker bonding between Gly and the Au surface.

The intrinsically dissimilar charge density differences and charge transfer between the S and N atoms explain their different energy-level movements in the PDOS upon adsorption, where the S atom exhibits significant peak broadening, whereas the N atoms show mainly peak shifts and slightly broadened areas. Our combined analysis of the adsorption of amino acid analogues on low-Miller-index facets paves the way for rationally designing the initial configurations to efficiently search for the most stable adsorbed state of L-GSH on Au(321) and to probe the enantiospecific binding.

### L-GSH Adsorption on Chiral Au(321)^*R*/*S*^ Facets

To evaluate whether L-GSH enantioselectively adsorbs on one of the Au(321)^*R*/*S*^ facets to promote the growth of 432 helicoids II and III, one typically needs to perform a comprehensive search for the most stable adsorbed states on *R* and *S* chiral surfaces, compare their binding energies to evaluate the enantiomeric shifts, and arrive at a conclusion on the likely thermodynamic influence. Such a search is relatively less computationally demanding for small molecules on simple facets, as was done in the first part of this work. However, the degrees of freedom associated with a full L-GSH molecule and kinked Au(321) make a brute-force geometric scan prohibitively expensive. Instead, by understanding how each amino acid building block of L-GSH interacts with the constituent microfacets of chiral Au(321), we can rationally design initial configurations that are likely to be near the ground-state energy, making the search for thermodynamically favored adsorption more efficient and cost-effective.

Combining the above discussion on the adsorption behaviors of amino acid analogues on low-Miller-index facets and previous studies on enantiospecific adsorption of organic molecules on surfaces with kinked sites,^17,28,29^ we propose a few initial configurations of L-GSH on both Au(321)^*R*^ and Au(321)^*S*^ for optimization based on the following rationale.

1. Starting with the Au(321)^*R*^ facet, the S atom is given the highest priority to be attached to a kink site on the surface and should be placed near the short-bridge site on the {110} step.
2. When orienting L-GSH on the surface, the N atom in the primary amine (N1 in Figure 1b) is given the second-tier priority to attach to another kink site. The N atom from Gly (N3 in Figure 1b) is considered next in a similar fashion. This is not only because the N atom in l-Glu shows a more significant contribution to binding than that in Gly from the PDOS analysis but also because it remains as a primary amine on a L-GSH, whereas the other N atoms join the peptide bond.
3. The left N atom (N2 in Figure 1b) in the backbone and all of the O atoms should be placed as close to the surface atoms as possible.
4. The binding energy of the same geometry relative to the Au(321)^*S*^ enantiomer is evaluated by flipping the L-GSH molecule 180° as indicated in Figure S12 and placing it onto the binding sites corresponding to Au(321)^*R*^.

Six sets of configurations on Au(321)^*R*/*S*^ were optimized using DFT, and the top views of all optimized configurations, along with the binding energy *E*_B_, are reported in Figure S12 in the Supporting Information. The binding energies *E*_B_ range from −2.063 to −2.839 eV and tend to cluster near the boundary values: five configurations have *E*_B_ close to −2.063 eV, six are close to −2.839 eV, and only one has a value in between. Almost all optimized geometries extend L-GSH longitudinally and orient it along the step edge to interact with multiple kink sites, similar to what was postulated by Lee et al.^17^ Although the most thermodynamically stable adsorption (*E*_B_ = −2.839 eV) is found to be on Au(321)^*R*^, there is no clear enantioselectivity between the *R* and *S* facets, for the second (*E*_B_ = −2.782 eV) and the third (*E*_B_ = −2.736 eV) most stable configurations are both on Au(321)^*S*^. Additionally, the preferences of the *R*- and *S*-facets are inconsistent across all pairs. Each pair was designed to test enantioselectivity with L-GSH in a similar geometry and orientation. Three pairs show a selectivity toward Au(321)^*S*^ (sets 2, 3, and 5), one pair strongly favors Au(321)^*R*^ (set 4), while two pairs exhibit nearly comparable binding energies (sets 1 and 6). The modest enantiomeric shift in adsorption observed in our results is consistent with that of previous work. It is also worth noting that the preference for the *S*- or *R*-facet depends not only on the completeness of the geometric scanning but also on the functional used. We have summarized the key findings from earlier first-principles studies on the enantiospecific adsorption of ligands on chiral facets in the SI.

Despite the subtle preference in the enantiomeric facets, the six configurations with *E*_B_ close to −2.8 eV (i.e., sets 1R, 1S, 2S, 3S, 4R, and 5S) share common geometric features regarding how L-GSH correlates with kink sites on Au(321)^*R*/*S*^. Here, we take the configuration with the strongest binding, corresponding to the (*R*)-enantiomer in set 4 in Figure S12, to illustrate. Its configurations and DOS analyses are listed in Figure 5.

**Figure 5:**
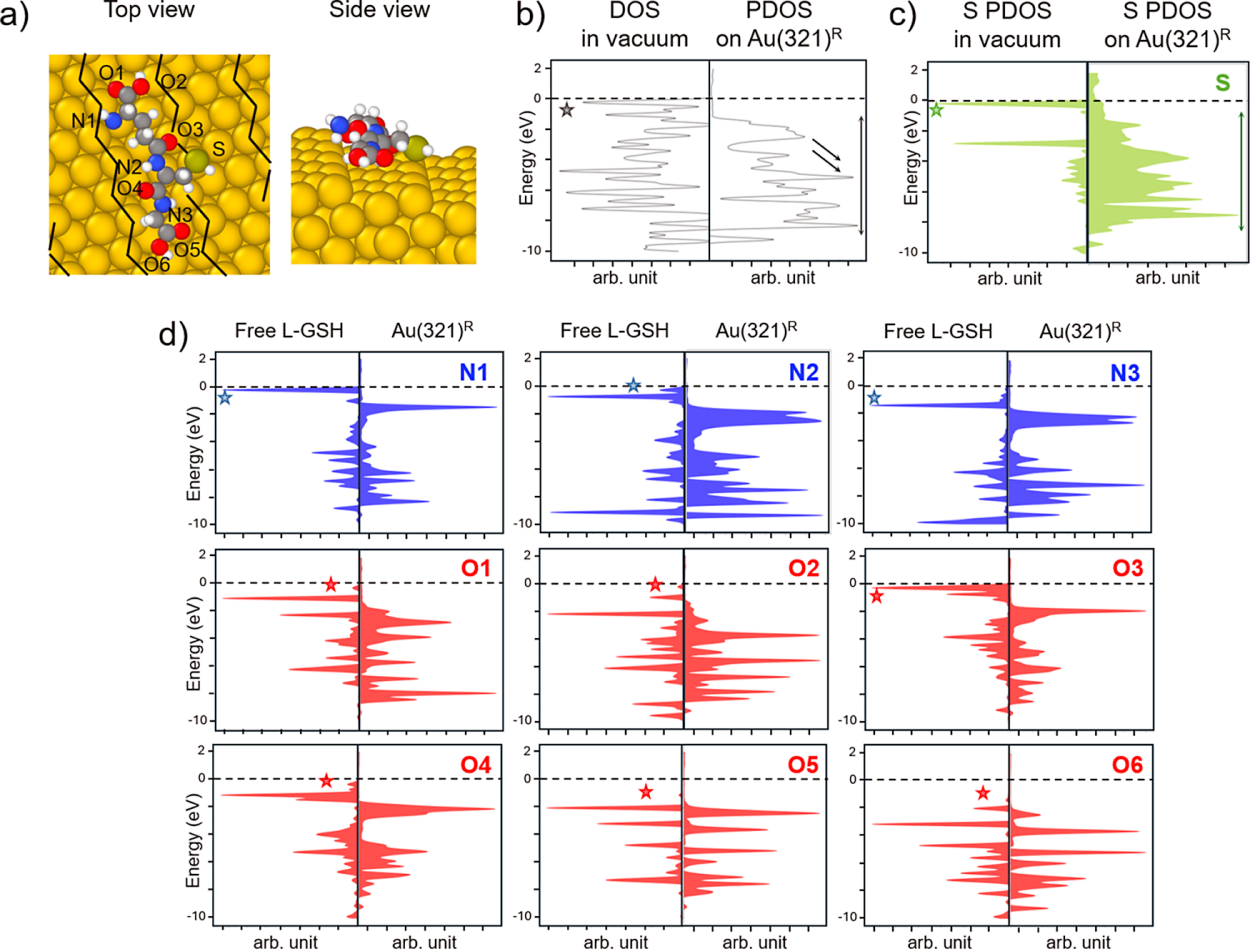
(a) Top view and side view of the adsorption configuration of L-GSH on Au(321)^*R*^, with black lines contouring the kinks. (b) DOS of an isolated L-GSH on the left and the PDOS of the adsorbed L-GSH molecule in (a) on the right. (c) PDOS on the S atom in an isolated L-GSH molecule on the left and in the adsorbed L-GSH molecule in (a) on the right. (d) PDOS on all N (blue) and O (red) atoms in an isolated L-GSH molecule on the left and in the adsorbed L-GSH molecule in (a) on the right. The Fermi level is set to energy zero and marked with dashed lines. The colored stars represent the HOMO peak. The single-headed arrows show the PDOS peak shifts, and the double-headed arrows show the broadening states.

Given the small size of a single Cys, previous studies on the chiral recognition of D- and l-Cys on chiral Au surfaces suggest that Cys “clips” onto the kink site like a “claw.”^28^ This “claw-like” geometry has the thiolate S close to the short-bridge site and the N atom on an atop or off-top site near the edge of the top {111} terrace, while leaving both O atoms on the lower {111} terrace. Interestingly, in our study, when the thiol group is embedded in a flexible molecule and placed next to a kinked site, it does not chemically transform into thiolate as we have seen in the l-Cys adsorption on Au(110) in Figure 2. In this case, the S atom in the thiol group is more likely to shift close to the {111} terrace after optimization, even if initially placed close to the short-bridge site on the {110} step. As the “true” short-bridge site in between the step and lower terrace becomes less accessible and the neighboring N, now part of the backbone, is unable to accept a proton, the thiol prefers to move upward onto the {111} terrace, and the S atom tends to settle on an off-top site in configurations with stronger binding. Such a site preference of the S atom is clearly shown in the top view of Figure 5a. Also, as the N atom in l-Cys joins the backbone, the “claw-like” feature in single Cys adsorption is lost, and now all of the heteroatoms on the Cys block (S, N2, and O4) of L-GSH are situated on the same terrace.

Another key feature common to the strong-binding configurations is that, in addition to interacting with multiple kink sites, L-GSH also extends laterally to engage with multiple steps, with N1 and the S atoms pointing in opposite directions, coordinating with kink sites at different terrace levels. The S atom in most optimized configurations is found at an off-top site on the edge of the {111} terrace, as expected from Figure 2. The preferred sites of N1 may vary, but they all tend to coordinate with the step edge of another level than the one the S atom binds to. The resulting stretched conformation of L-GSH naturally positions the other heteroatoms on the {111} terrace, including all O atoms, N2, and N3. We denote this binding behavior in a flexible molecule, in contrast to a smaller, more rigid Cys, as a “reach-and-stretch” mechanism, in which a stretch of the entire molecule results from the reach of the key heteroatoms to their preferred sites. We also included top and side views of the relaxations of Sets 4R and 5S for clearer illustration (see Movies S1, S2, S3, and S4). Such behavior is consistent in all strong-binding configurations. When N1 and S atoms do not reach across two levels of steps, the binding will be significantly weaker, even when interacting with multiple kink sites along the same step (see Sets 2R, 3R, 4S, 6S, and 6R in the SI).

The total DOS of an isolated L-GSH molecule and the PDOS of the adsorbed L-GSH in the configuration shown in Figure 5a are shown in Figure 5b, and the PDOS of all heteroatoms are included in Figure 5c,d. In addition to the disappearance of the HOMO peak, marked with a star, the PDOS of the adsorbed L-GSH exhibits substantial peak broadening (double-headed arrow) and considerable peak shifts (single-headed arrow). By comparing the shapes of PDOS in Figure 5b and the right panel in Figure 5c, we conclude that the peak broadening between energy levels −3 and −9 eV in Figure 5b is mainly attributed to the S atom, which can be expected from the previous discussion on amino acid analogues.

Contrary to Figure 3, N in the Glu block (N1) shows the least movement of energy levels upon adsorption, while N in the Cys block (N2) exhibits the most noticeable peak broadening, followed by N in the Gly block (N3). This indicates that backbone N atoms contribute more significantly to overall binding than the primary amine. In fact, the broadened peaks from energy levels −1 to −3 eV help explain the peak broadening at the same energy levels in Figure 5b, alongside the contribution from the S atom. We argue that the greater contribution of the backbone N is highly correlated with the laterally stretched L-GSH conformation, where heteroatoms close to the backbone are pushed toward the Au(111) terrace, thus enhancing electron transfer to the Au slab. Although not directly contributing to binding via electron redistribution, the amine still plays an important role in “reach-and-stretch”, enabling maximized contact between the molecule and the surface.

Furthermore, we find that the PDOS of the O atoms in L-GSH displays more obvious peak broadening compared to the amino acid results. This, again, is due to the laterally stretched configuration that brings the O atoms closer to the surface than those in unconnected amino acids (Figure 2). Therefore, although they serve different roles, all heteroatoms on the L-GSH molecules collectively contribute to strong binding on a chiral Au(321) facet.

In shape-controlled synthesis of inorganic nanocrystals, it has long been believed and repeatedly demonstrated that facet-selective ligand binding, or structure-directing agents, promotes the expression of preferred facets.^55–57^ The facet-selective binding is manifested thermodynamically as stronger binding to a specific facet than to the others.^58^ However, these were tested and concluded from studies focusing on Wulff structures or relatively simpler polygons where the low-Miller-index facets were mostly involved. Here, we argue that knowledge of the adsorption behavior of a single ligand on a chiral surface in vacuum is insufficient to draw a definitive conclusion that enantioselective binding promotes the expression of the preferred facet. First, the facet evolution accompanied by the emergence of chiral structures remains an open question. Since the first introduction in 2018, the experimental understanding of the chiral growth mechanism has substantially evolved. Earlier studies argued that the chiral shape modifiers l-Cys and L-GSH prefer the *R* facets, leading to slower vertical growth on Au(321)^*R*^ and to the boundary between Au(321)^*R*/*S*^ extending from the R region to the S region.^17,19^ With more comprehensive examination of the chiral gap structure and time-resolved analysis on chiral growth, later findings suggest that such mechanistic explanations are more likely historical hypotheses than definitive conclusions, as recent studies show that the S-type kinks are, in fact, more abundant on chiral gold nanoparticles synthesized with L-GSH, and the percentage of S-type kinks increases as growth proceeds.^59,60^

On the other hand, while the enantioselectivity of the ligand to the chiral facets depends on the choice of functional in first-principles calculations, the binding energy’s enantiomeric shift is generally small for various ligand and facet pairs (see the supplementary table in the Supporting Information). Also, in our study, while the strongest binding occurs on the *R* facet, four of the top six configurations with *E*_B_ close to −2.8 eV are on Au(321)^*S*^, with *E*_B_ ranging from −2.734 to −2.782 eV. When a strong enantiomeric shift in binding energies is lacking, which makes thermodynamic regulations less feasible as they often require disparate adsorption selectivity to significantly alter the interfacial free energies of various facets,^61^ we lack direct evidence to decisively arrive at the conclusion that the facet preferred by a single molecule in vacuum determines which chiral facet will be predominantly expressed in the final outcome.

With such a subtle enantiomeric shift in binding energies, we postulate that the influence of L-GSH in promoting 432 helicoid II and III is most likely via kinetic regulation and largely associated with their adsorbed configurations on the facets. In addition to the laterally stretched conformation of L-GSH, we find that the two stable L-GSHs on Au(321)^*R*/*S*^, respectively, position the backbone at distinct sites. For Au(321)^*R*^ in Figure 5, the backbone is aligned against the bottom of the step edge, whereas for Au(321)^*S*^ (i.e., set 5S in Figure S12), the backbone is aligned along the top of a step edge. The difference in the preferred locations of the backbone may provide channels to guide adatom migration and promote the kinetic growth of a certain facet kinetically.

## Conclusions

We have designed an efficient and cost-effective scheme to examine the adsorption of L-GSH on Au(321)^*R*/*S*^ using first-principles DFT. We first decomposed the full L-GSH molecule into l-Glu, l-Cys, and Gly amino acid analogues and the Au(321) facets into the constituent microfacets Au(111), Au(100), and Au(110). Extensive configuration scans were conducted for each amino acid and low-Miller-index pair, including identification of the key heteroatom, rotation of the molecule about different principal axes, and translation of the molecule among typical binding sites. For each pair, we identified the most stable adsorbed state with the lowest *E*_B_ value and performed DOS and PDOS analyses on the isolated molecules and adsorbates. A detailed examination of all adsorbed configurations indicates that the S atom in l-Cys and the N atoms in l-Glu and Gly primarily contribute to binding. Their critical roles are confirmed by PDOS analysis, charge density difference plots, and Bader charge-transfer calculations, which reveal the most pronounced hybridization with Au electrons for the S atom, moderate hybridization for N atoms, and the least for O atoms. The top site is most energetically favorable for the N atoms in l-Glu and Gly on Au(111) and Au(100). In the absence of chemical transformation, the off-top site is preferred by the S atom on Au(111) and Au(100) and by both N atoms on Au(110). Neutral l-Cys with a thiol group transforms into an unconventional zwitterion on Au(110), where the short-bridge site becomes the preferred site for S. For all low-Miller-index facets, the molecular binding affinity ranks as l-Cys > l-Glu > Gly, with *E*_B_ significantly more negative for l-Cys on Au(110) due to the shift from thiol to thiolate. Note that the thiol group remains after optimization of Au(111) and Au(100). Moreover, the facet preference follows Au(110) > Au(100) > Au(111). The selectivity toward Au(100) over Au(111) could be a contributing factor to the formation of the cubic outline on 432 helicoid III.

Findings from the analysis of amino acid building block binding on microfacets serve as the rationale for designing the initial configuration of L-GSH adsorbed on Au(321) for optimization. The design prioritizes bonding between the S atom and the primary amino-N atom in the Glu block at the kink sites. The other N atoms and all of the O atoms are positioned as close to the Au surface as possible at last. Six sets of L-GSH adsorbed on Au(321)^*S*^ and Au(321)^*R*^ were constructed and optimized. Our decomposition-based strategy has efficiently and successfully explored the ground-state adsorption of flexible L-GSH on complex chiral surfaces, demonstrating a powerful and transferable computational framework for addressing ligand adsorption that would otherwise be computationally prohibitive.

Among the 12 optimized configurations, the most thermodynamically stable adsorption corresponds to an L-GSH on Au(321)^*R*^, but the preference for the (*R*)-enantiomer is relatively weak, as the enantiomeric shift of *E*_B_ is subtle. Our finding of moderate enantioselectivity aligns with previous DFT results.^23,62,63^ By comparing all configurations with strong-binding affinity, we find that L-GSH not only interacts with multiple kink sites, as postulated by Lee et al., but also coordinates with multiple steps via the amine and thiol groups. By reaching close to the steps at different terrace levels, L-GSH is laterally stretched. This “reach-and-stretch” effect maximizes contact of all heteroatoms with the {111} terrace atoms, including the O atoms, which were shown to be less significant on free-standing amino acids. This is crucial for stabilizing the chiral facets. Our approach to rationally identifying stable adsorption states of large molecules on complex facets can inspire similar studies in the future. Meanwhile, some conflicting findings between amino acid and L-GSH studies highlight the importance of the configurational degrees of freedom of large molecules; therefore, caution should be exercised when predicting long-chain behavior from their building blocks.^64^

Conventionally, in shape-controlled nanocrystal synthesis, it is believed that when binding selectively to a certain facet, the ligand promotes the expression of that facet in the final shape outcome. On the basis of the most recent experimental progress, the earlier hypothesis that a preference for L-GSH on the *R* facets shifts from the R region to the S region has now become obsolete, as more advanced characterization methods have shown that the S-type kinks are more abundant in the final structure. While the enantiomeric selectivity of L-GSH Au(321)^*R*^ conforms with previous DFT studies on Cys, we find that the local energetic preference obtained in the gas phase alone, especially when the enantioselectivity is moderate, is insufficient to thoroughly explain how the ligand influences colloidal nanoparticle growth on a more global scale.^17,19,59,60^ Disentangling the convoluted interplay between thermodynamic and kinetic effects requires more systematic studies that explicitly consider solvation, temperature, a full adlayer of L-GSH and other ligands, and Au surface adatom dynamics. For ligands with weak to moderate facet selectivity, thermodynamic control is less likely. When the local energetic preference does not align with the overall facet expression, the kinetics of surface adatom diffusion, such as step-flow growth, imparted by the ligand adlayer, becomes more critical to understanding the chiral growth mechanism. These events often involve dynamics and configurations other than the ground states in solvated environments with additional chemical additives, which are beyond the reach of the DFT calculations. Our work contributes to an initial effort to uncover the roles of the chiral shape modifiers. Multiscale approaches that combine all-atom molecular dynamics simulations with rigorously derived force fields, Monte Carlo, and kinetic Monte Carlo simulations should be used in future studies to enable comprehensive theoretical investigations.^65,66^

## Supplementary Material











## Notes

Au slab optimization, additional adsorption configurations of amino acid analogues on microfacets, PDOS analysis on less important heteroatoms, optimized adsorbed configurations of L-GSH on Au(321)^*R*/*S*^, a summary table of previous DFT works, and videos showing L-GSH relaxation (PDF)
Top view of the relaxation of Set 4R (MP4)
Side view of the relaxation of Set 4R (MP4)
Top view of the relaxation of Set 5S (MP4)
Side view of the relaxation of Set 5S (MP4)
