# Supplementary material for: A First-Principles Study of l‑Glutathione Adsorption on Chiral Gold Nanoparticles
Source: ACS Mater Au. 2026 May 25;6(4):767–78. doi: 10.1021/acsmaterialsau.5c00262 (PMC13352273; doi:10.1021/acsmaterialsau.5c00262)
Supplement: Supplementary file 1 [file mg5c00262_si_001.pdf]

Supporting Information: A First-principles Study of L-glutathione Adsorption on Chiral Gold Nanoparticles

Jianming Cui[1] and Xin Qi*[1]

[1]*Department of Chemistry, Dartmouth College, Hanover, NH 03755, USA*

*Email: xin.qi@dartmouth.edu

Au Slab Setup

The optimized lattice constant of Au was found to be 4.10 Å, which is in good agreement with the experimental value of ~4.07 Å (Figure S1(a)). [1-3] The total DOS of the Au(321) slab is shown in Figure S1(b), where the Fermi level is set to 0. The energy level ranges from -2 to -6 eV represents the Au *d* orbital, which is consistent with and suggested by previous studies. [4, 5]

The Au(321) slab setup is not as straightforward as the low-Miller-index facets, given its kinked steps. We find it necessary to prepare a thick enough slab to avoid surface reconstruction (additional trial data not shown). As shown in Figure S1(c), an initial six-layer Au(321) slab was used for relaxation, and the top four layers were retained for subsequent adsorption calculations.

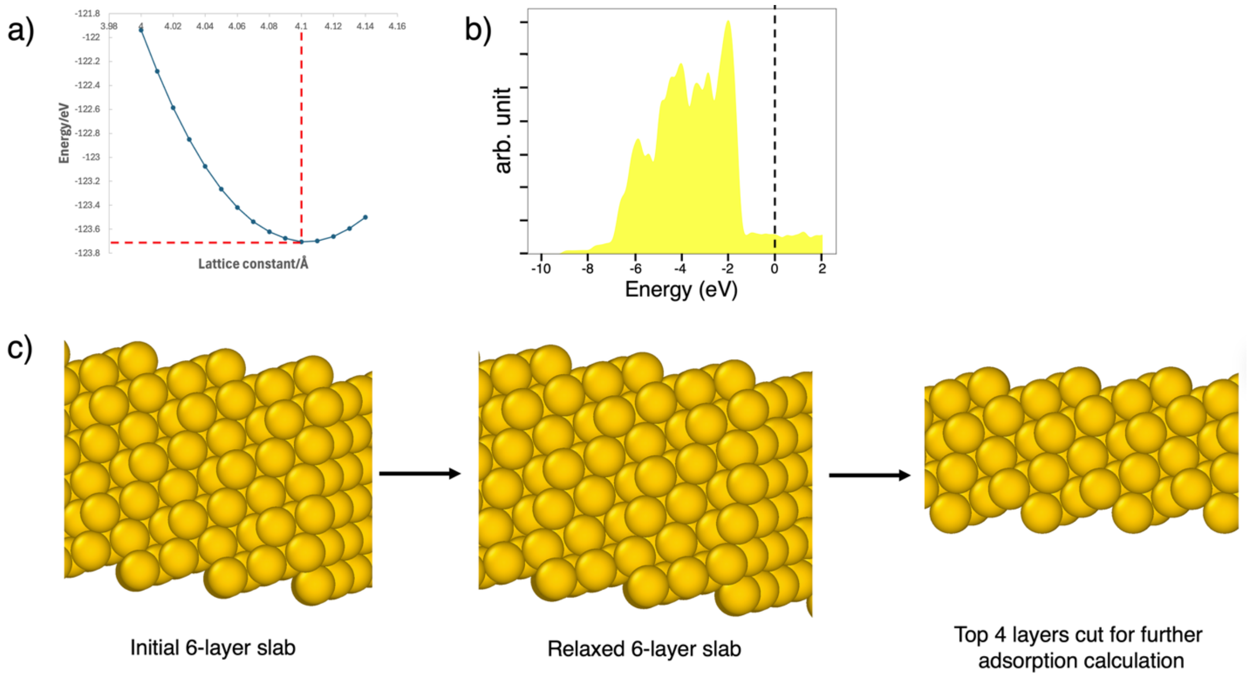

Figure S1: a) The optimal lattice constant for Au is identified as the lattice constant that yields the lowest total energy of a 2 × 2 × 2 Au bulk with a k-point mesh of 6 × 6 × 6. b) Total DOS plot of an Au(321) slab. The Fermi level is set as zero, marked with a black dashed line. c) Scheme of Au(321) setup. A six-layer Au(321) slab is prepared for relaxation, and the top four layers are kept and fixed for further use.

Additional Adsorption Configurations

In the next nine figures, we show three additional adsorption configurations for each amino acid analogue (L-Cys, L-Glu, and Gly) and low-Miller-index facet (Au(100), Au(111), and Au(110)) pairs. For each adsorption configuration, we show both the initial and relaxed (i.e., final) configurations. Their atom position shift during relaxation indicates the preferred locations of each atom relative to the Au surface atoms. The general trend for each pair is discussed in the caption.

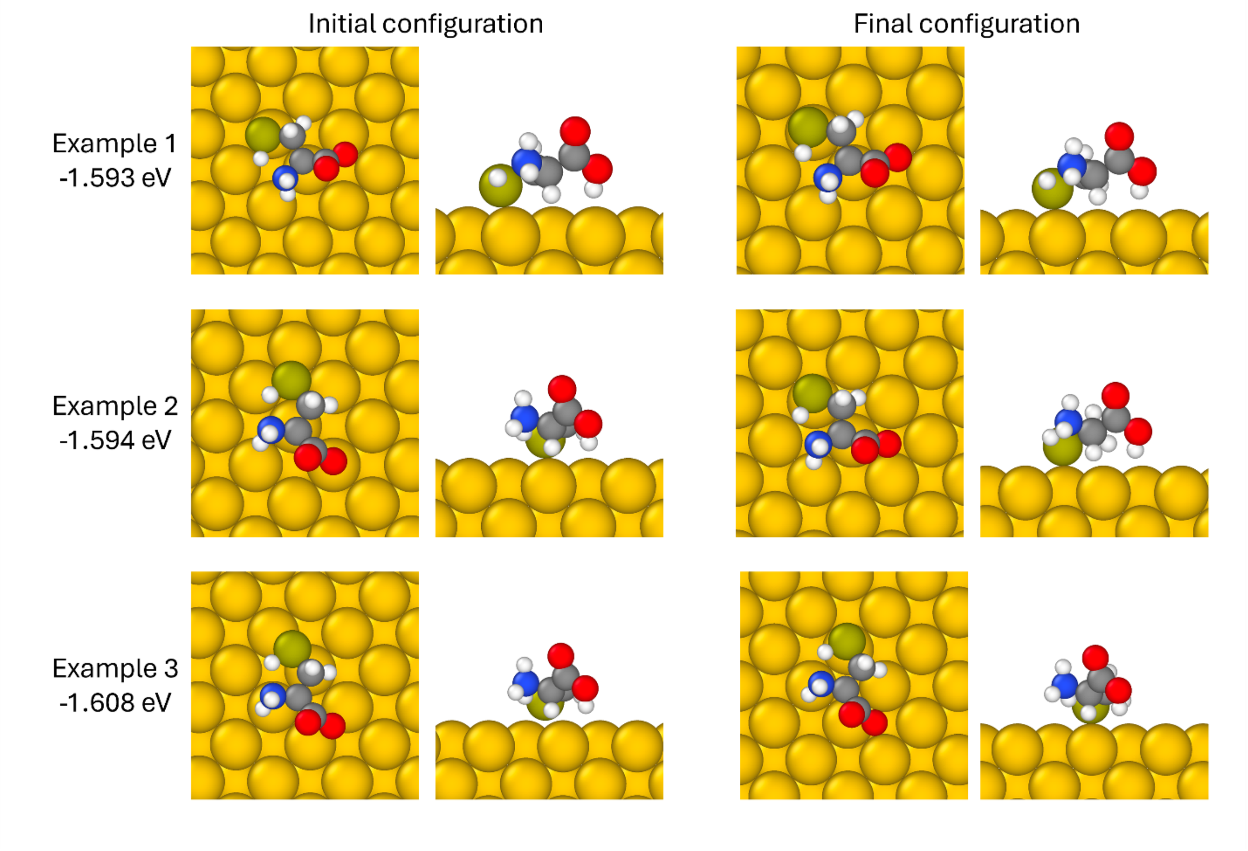

Figure S2: Three additional adsorption configurations, including both initial and final images of **L-Cys on Au(100)**, viewed from the top and side, along with the binding energies. The C atoms are shown in grey, O atoms in red, N atoms in blue, S atoms in lime, H atoms in white, and Au atoms in gold. In these examples, the most important heteroatom, S, is tested for location on the off-top, hollow, and bridge sites. All optimized configurations exhibit that the S atom prefers the off-top site.

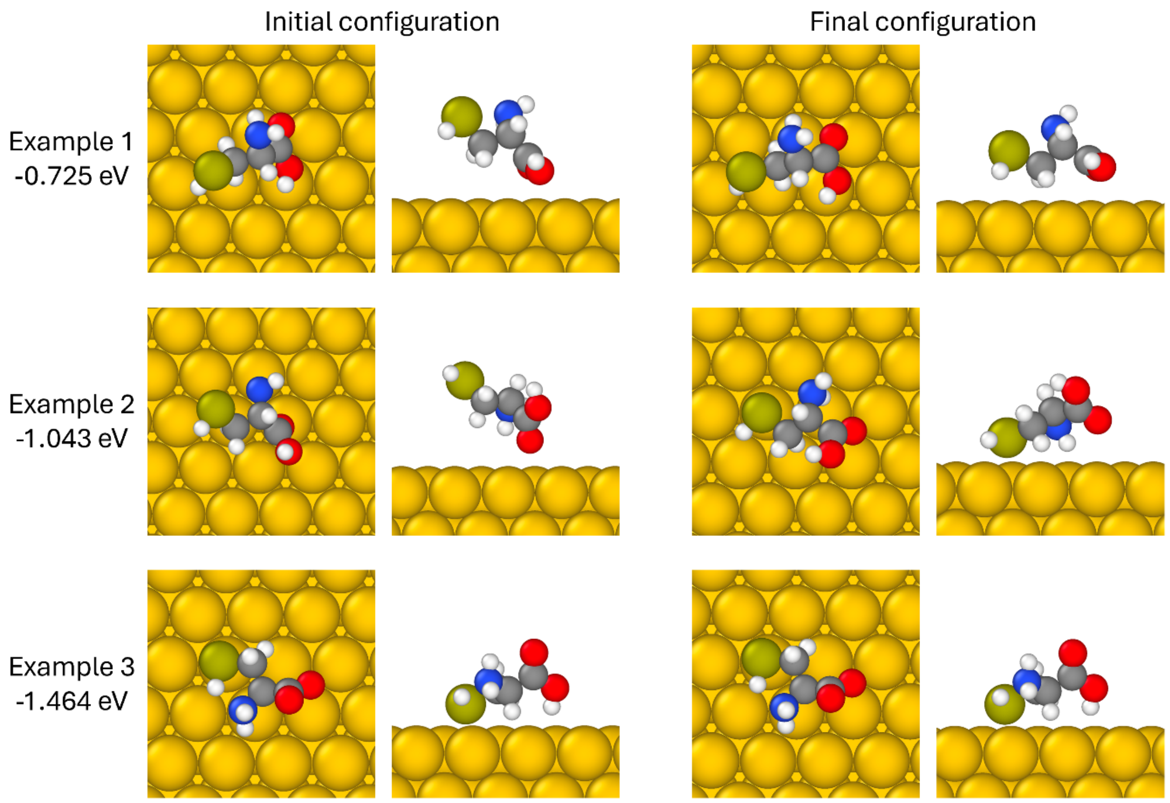

Figure S3: Three additional adsorption configurations, including both initial and final images of **L-Cys on Au(111),** viewed from the top and side, along with the binding energies. The C atoms are shown in grey, O atoms in red, N atoms in blue, S atoms in lime, H atoms in white, and Au atoms in gold. In these examples, initial configurations with different heteroatoms approaching the Au surface are tested. The optimized configuration in example 1 places the S atom furthest from the Au surface and exhibits the weakest binding, indicating that the S atom is the most important heteroatom and should be prioritized when designing the configuration setup for L-GSH on Au(321).

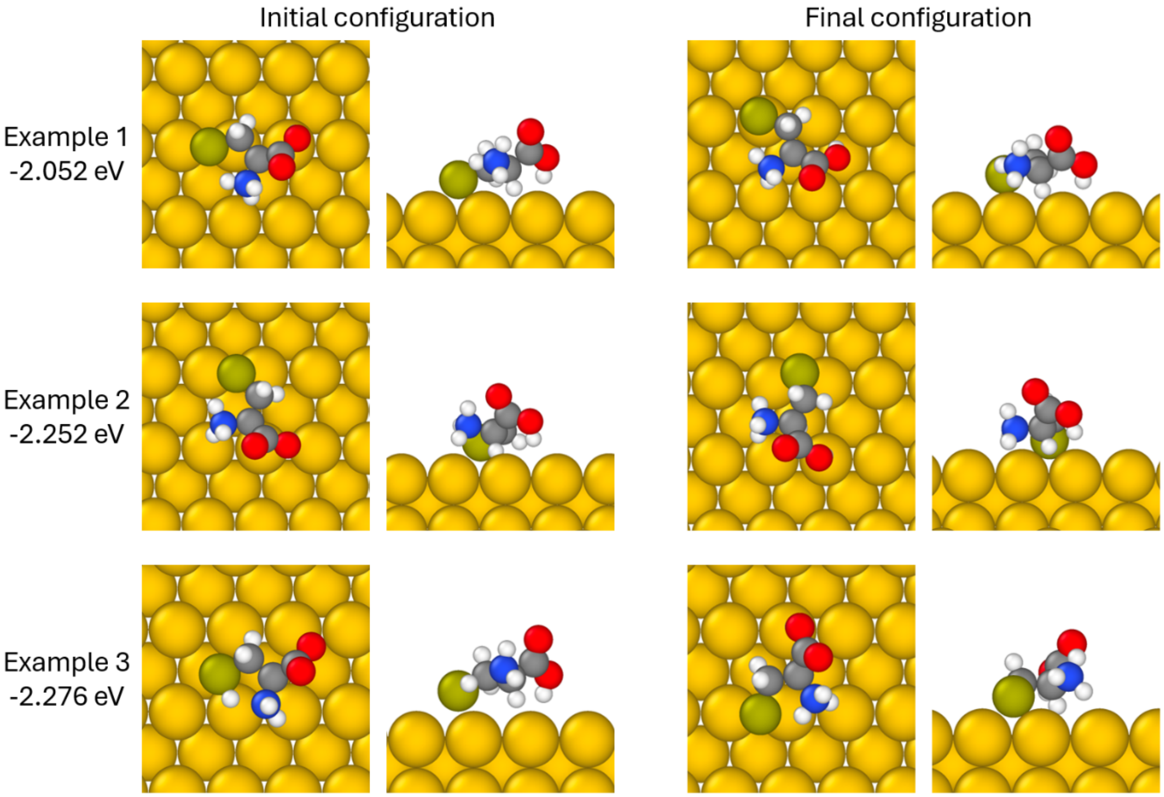

Figure S4: Three additional adsorption configurations, including both initial and final images of **L-Cys on Au(110),** viewed from top and side, along with the binding energies. The C atoms are shown in grey, O atoms in red, N atoms in blue, S atoms in lime, H atoms in white, and Au atoms in gold. In these examples, the S atom was initially located at the short-bridge site (Examples 1 and 2) with different angles and at the long-bridge site (Example 3). Although starting with S at different sites, Examples 2 and 3 both ended with S at the short-bridge site, suggesting the short-bridge site is preferred over the long-bridge site. Despite the N atom being closer to the surface in Example 2, Example 3 shows slightly stronger overall binding, suggesting that the short-bridge site is also a preferred site for N, which is consistent with what has been found in the configuration shown in Figure 2.

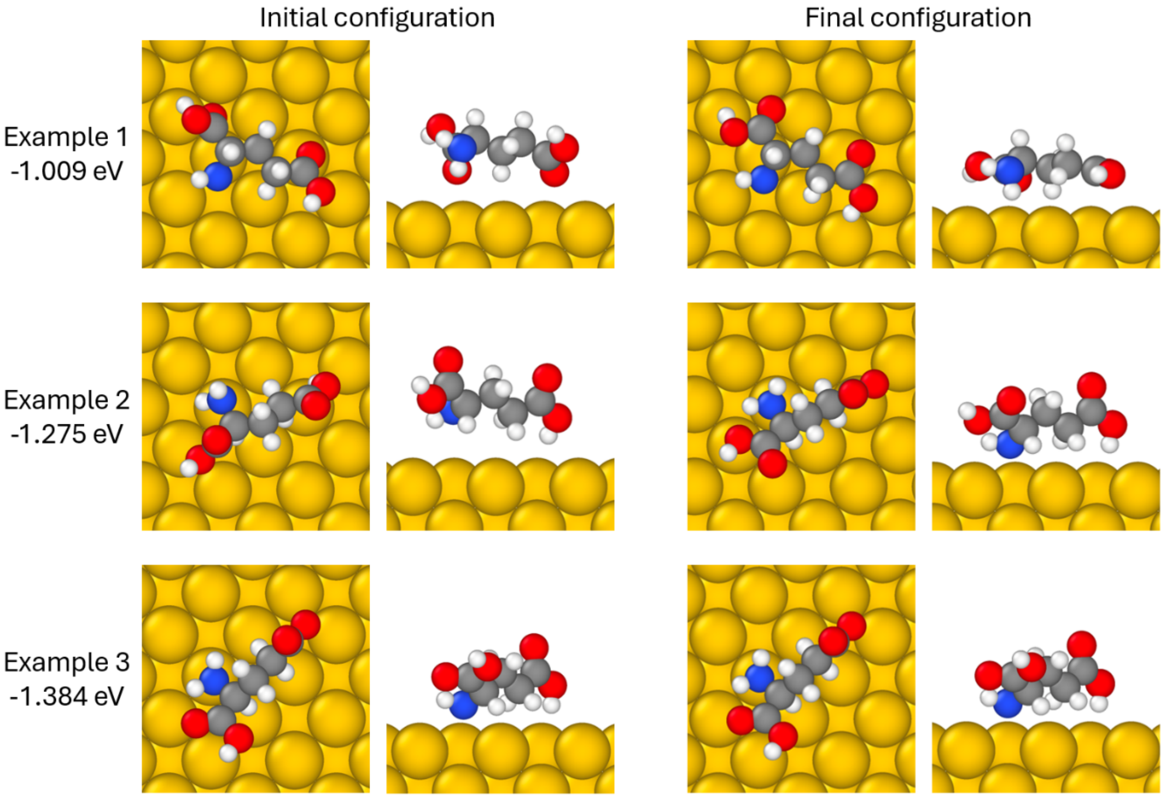

Figure S5: Three additional adsorption configurations, including both initial and final images of **L-Glu on Au(100),** viewed from top and side, along with the binding energies. The C atoms are shown in grey, O atoms in red, N atoms in blue, S atoms in lime, H atoms in white, and Au atoms in gold. In these examples, initial configurations with different heteroatoms approaching the Au surface are tested. The correlation between the binding energy and the distance between the atom and the Au surface suggests that the N atom is critical in determining the binding energy. In contrast, the O atom getting closer to the Au surface does not guarantee a stronger binding (c.f. Example 1 and Examples 2 and 3). It is interesting to find that the backbone alignment with the bridge site (Example 2 vs Example 3 and Figure 2) also enhances the binding energy.

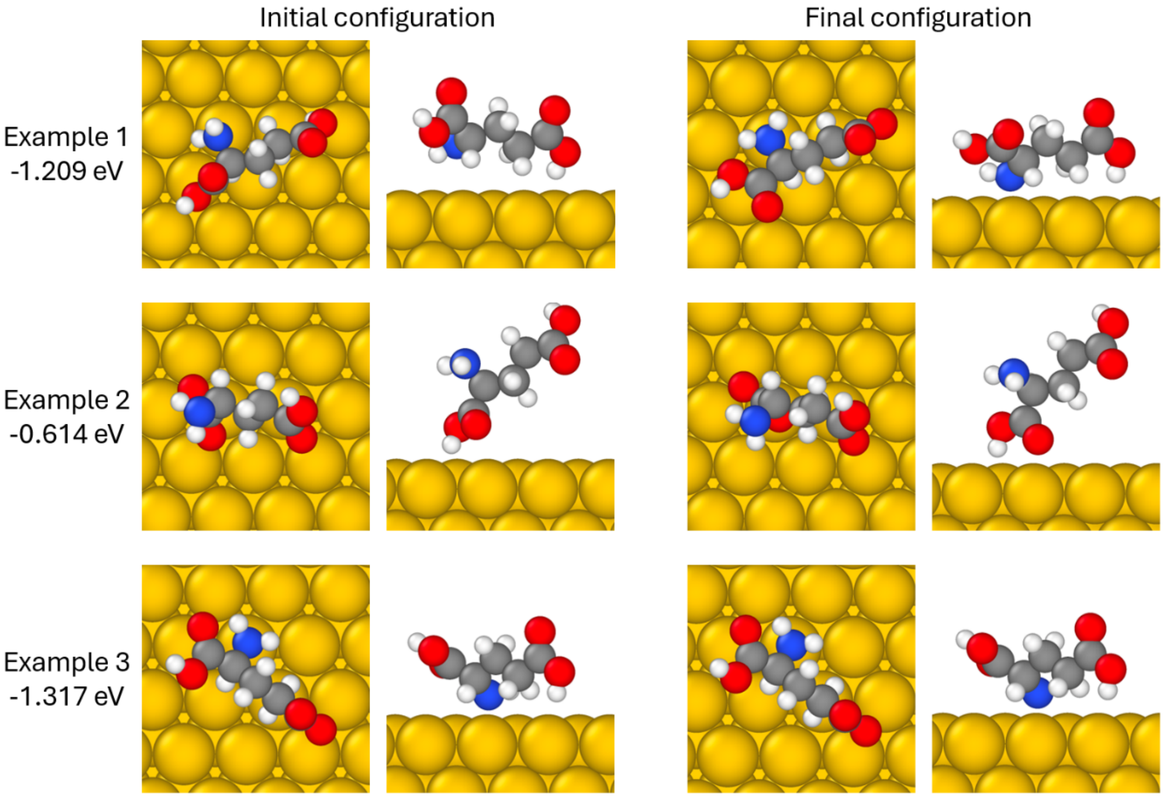

Figure S6: Three additional adsorption configurations, including both initial and final images of **L-Glu on Au(111),** viewed from top and side, along with the binding energies. The C atoms are shown in grey, O atoms in red, N atoms in blue, S atoms in lime, H atoms in white, and Au atoms in gold. In these examples, initial configurations with different heteroatoms approaching the Au surface are tested. It shows that the N atom should have the highest priority for locating at the optimized position, and then the O atoms and the backbone should lie on the Au surface rather than stand.

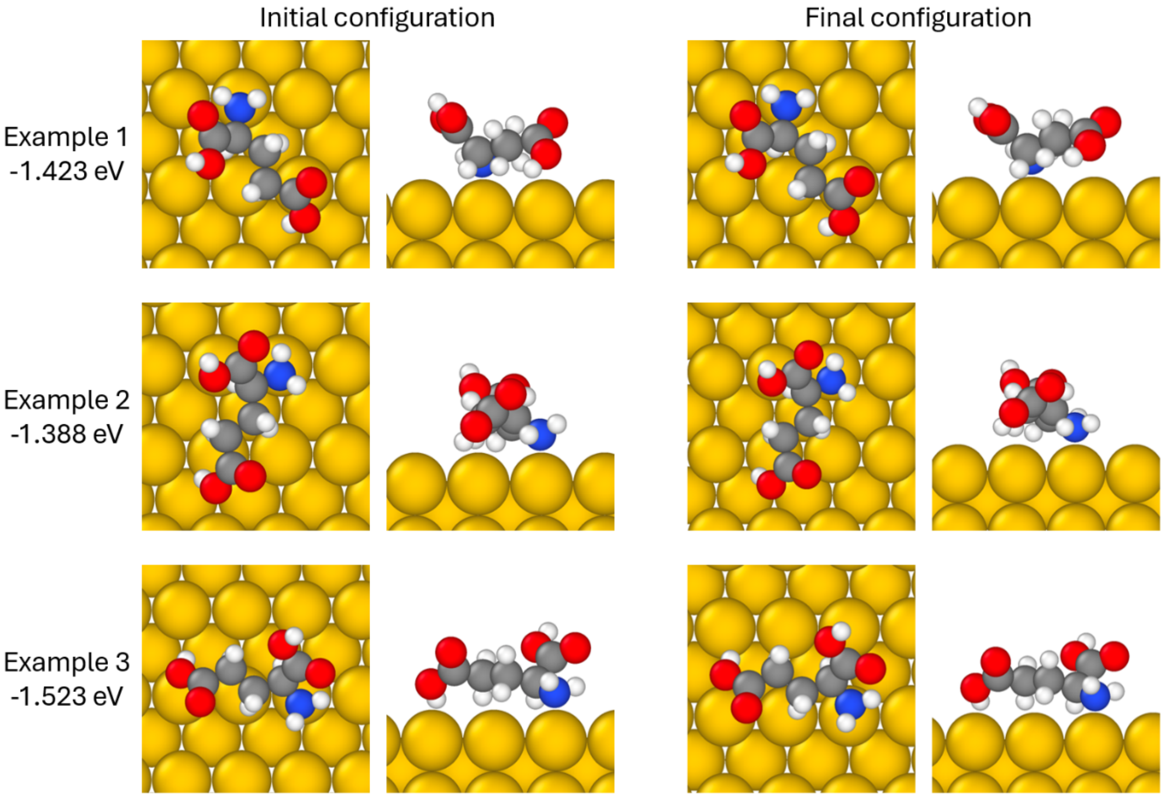

Figure S7: Three additional adsorption configurations, including both initial and final images of **L-Glu on Au(110),** viewed from top and side, along with the binding energies. The C atoms are shown in grey, O atoms in red, N atoms in blue, S atoms in lime, H atoms in white, and Au atoms in gold. In these examples, the N atom is located initially at the off-top site, and its location is relatively maintained during optimization. The optimization mainly involves a slight adjustment of the O atom orthogonal distance from the surface, which may involve shifting away from the surface (see Example 1). Both Figure 2 and Example 3 show that the C atoms prefer the long-bridge sites.

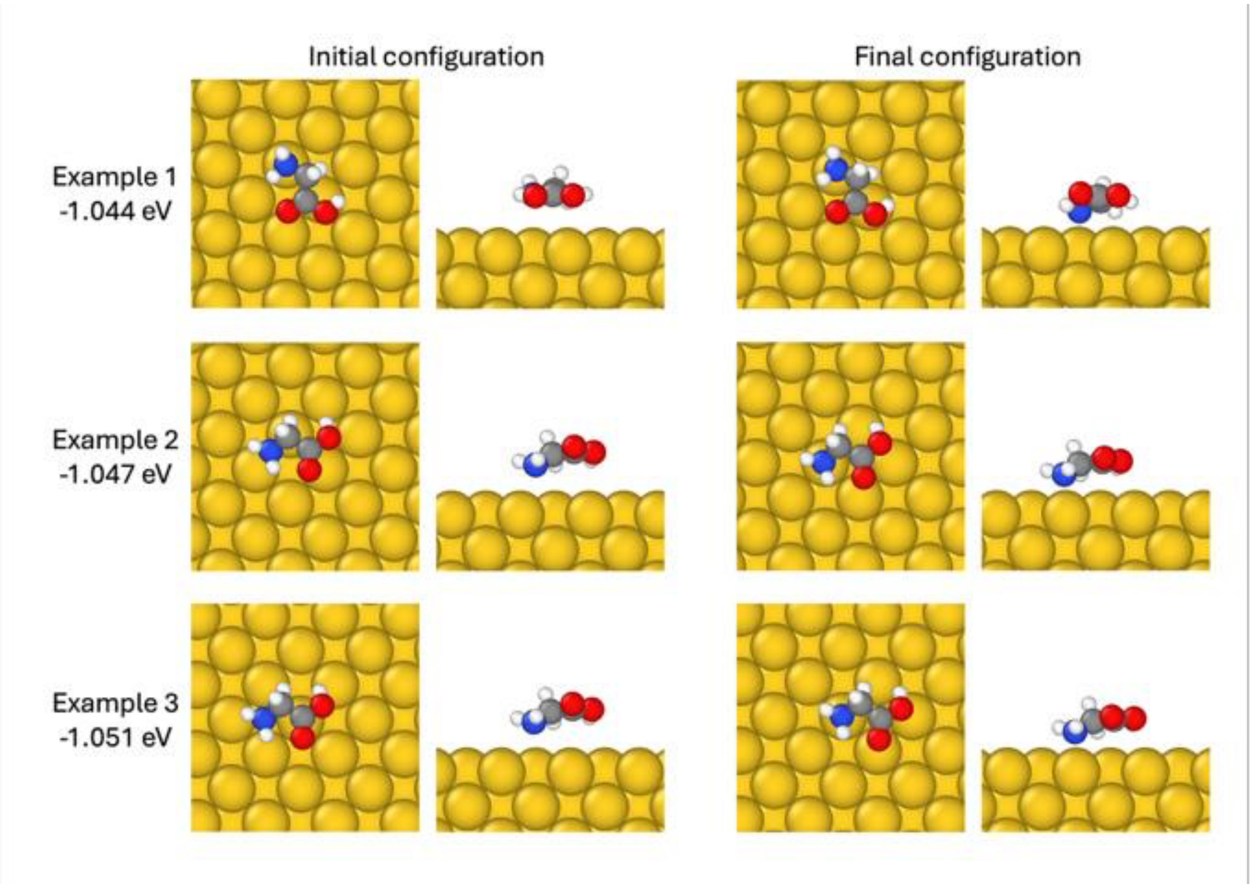

Figure S8: Three additional adsorption configurations, including both initial and final images of **Gly on Au(100),** viewed from top and side, along with the binding energies. The C atoms are shown in grey, O atoms in red, N atoms in blue, S atoms in lime, H atoms in white, and Au atoms in gold. In these examples, the N atom is located initially at the atop site (example 1), the bridge site (example 2), and the hollow site (example 3). The final configuration of all examples shifts the N atom to the atop site, consistent with Figure 2. The locations of other atoms play a minor role in determining the binding energy.

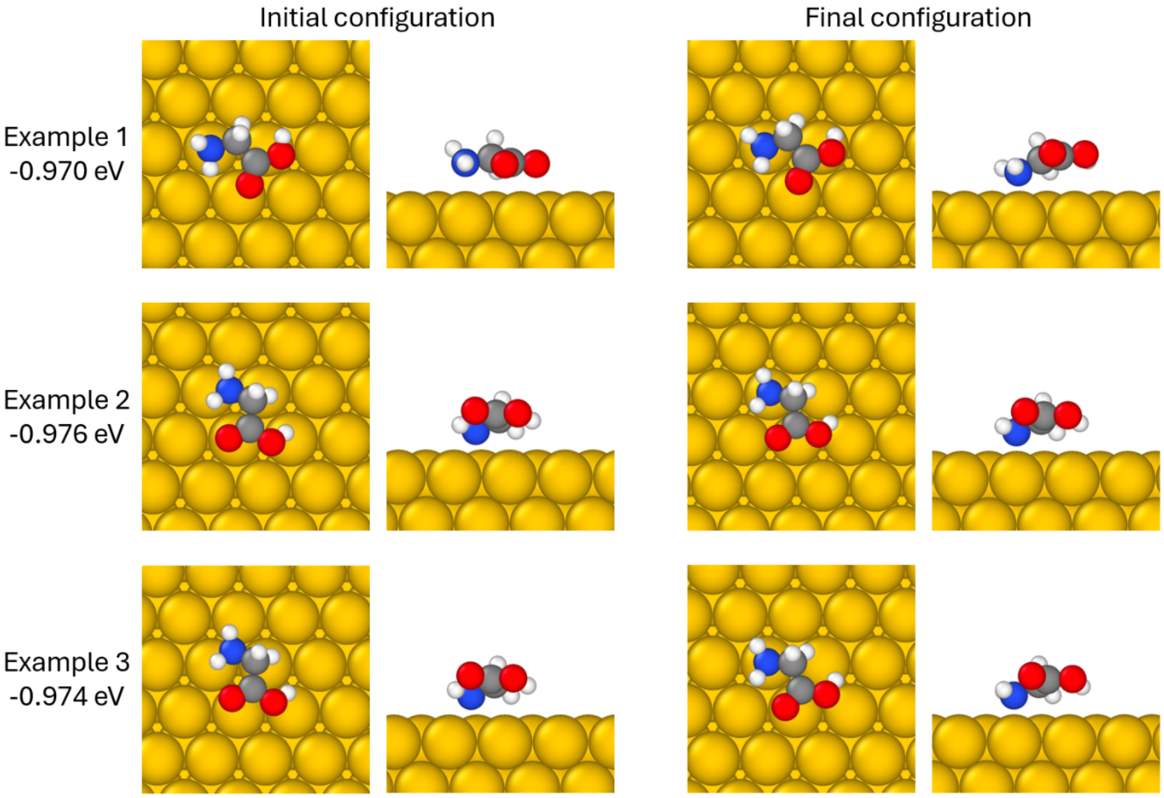

Figure S9: Three additional adsorption configurations, including both initial and final images of **Gly on Au(111),** viewed from top and side, along with the binding energies. The C atoms are shown in grey, O atoms in red, N atoms in blue, S atoms in lime, H atoms in white, and Au atoms in gold. In these examples, the N atom is located initially at the atop site (Example 1), the bridge site (Example 2), and the hollow site (Example 3). Similar to Figure S8, the final configuration of all examples shifts the N atom to the atop site, consistent with Figure 2. The locations of other atoms play a minor role in determining the binding energy.

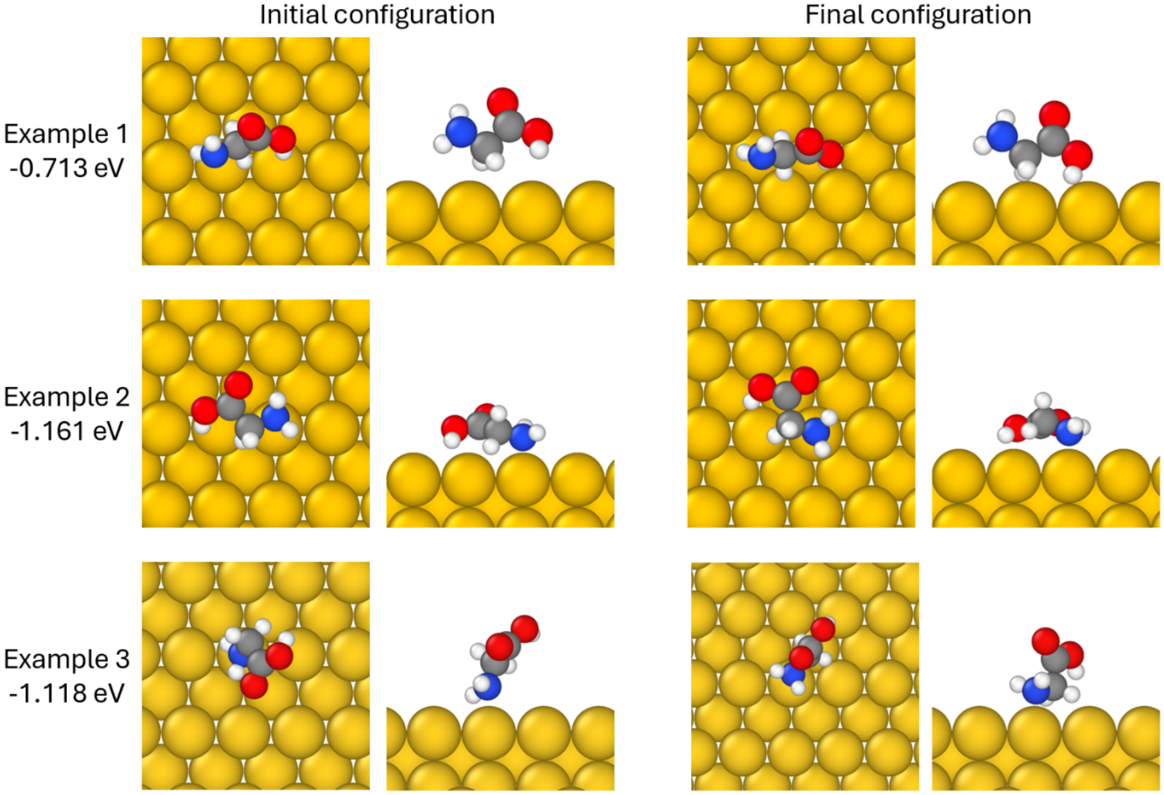

Figure S10: Three additional adsorption configurations, including both initial and final images of **Gly on Au(110),** viewed from top and side, along with the binding energies. The C atoms are shown in grey, O atoms in red, N atoms in blue, S atoms in lime, H atoms in white, and Au atoms in gold. In these examples, initial configurations with different heteroatoms approaching the Au surface are tested. Comparing Example 1 with others, we conclude that the N atom contributes most to binding. The orthogonal distance of the O atom also influences binding energy, but not as critically as the N atom (c.f. Examples 2 and 3). Finally, the backbone should lie down on the Au surface instead of standing.

PDOS of less important heteroatoms

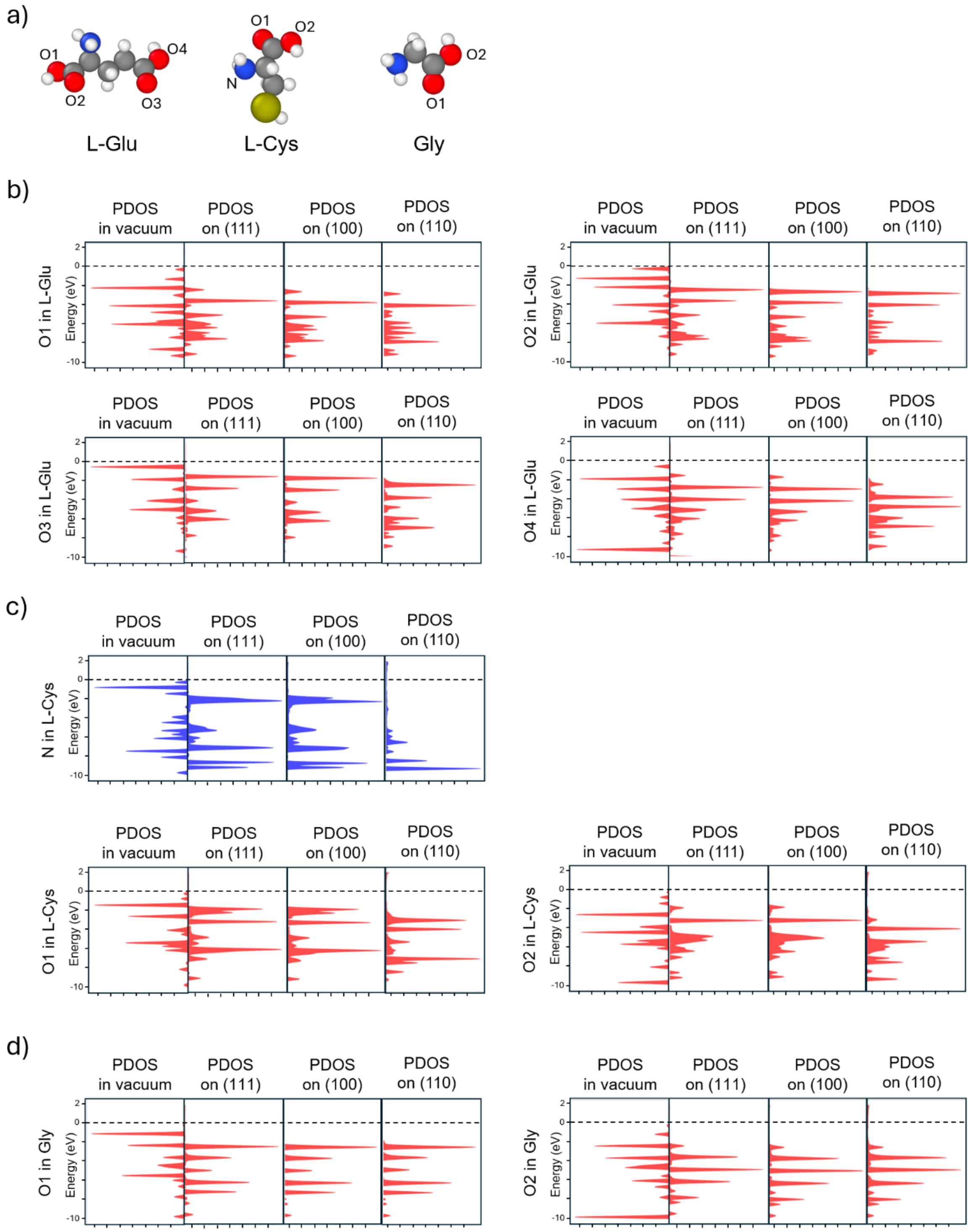

Figure S11: a) The molecular structures of L-Glu, L-Cys and Gly, with the less important heteroatoms (*e.g.,* N and O atoms) labeled for PDOS analysis and bond length calculation. The

PDOS of the less important heteroatoms of b) L-Glu, c) L-Cys and d) Gly. The leftmost panels refer to the isolated molecules in vacuum, and the right three panels refer to the adsorption configurations in Figure 2 in the main text. The Fermi level is set to zero, marked with black dashed lines.

Figure S11 (b)-(d) shows the projected density of states (PDOS) for less important heteroatoms (*e.g.*, N and O for L-Cys, and O for L-Glu and Gly), with the corresponding labels shown in Figure S11 (a). These less important heteroatoms reported in Figure S11 mainly exhibit peak shifting and slight broadening in the Au d orbital range from -2 to -8 eV. The disappearance of the HOMO peak, if there is, such as O1 and O2 in L-Glu, and N, O1 and O2 in L-Cys, is less notable compared to the most important heteroatoms reported in the main text, suggesting much weaker bonds with the Au surfaces, which served as the criterion to select the most important heteroatoms in the main text.

The bond lengths between the less important heteroatoms shown in Figure S11(a) and the Au surface are listed in Table S1, where the values are significantly larger than the bond lengths shown in Figure 2. The bond length comparison aligns well with the PDOS results, confirming the selection of the most important heteroatoms once again.

Table S1: Summary of bond length (Å) between less important heteroatoms and Au, D_{Au-x}.

		Au(111)	Au(100)	Au(110)
L-Glu	D_{Au-O1}	4.48	4.31	4.29
	D_{Au-O2}	3.93	3.89	4.81
	D_{Au-O3}	5.49	5.46	3.04
	D_{Au-O4}	3.62	3.68	3.14
L-Cys	D_{Au-N}	3.84	3.58	3.38
	D_{Au-O1}	5.29	5.04	5.00
	D_{Au-O2}	3.58	3.69	3.27
Gly	D_{Au-O1}	3.77	3.68	3.28
	D_{Au-O2}	3.41	3.32	3.38

Optimized L-GSH Adsorption Configurations on Au(321)

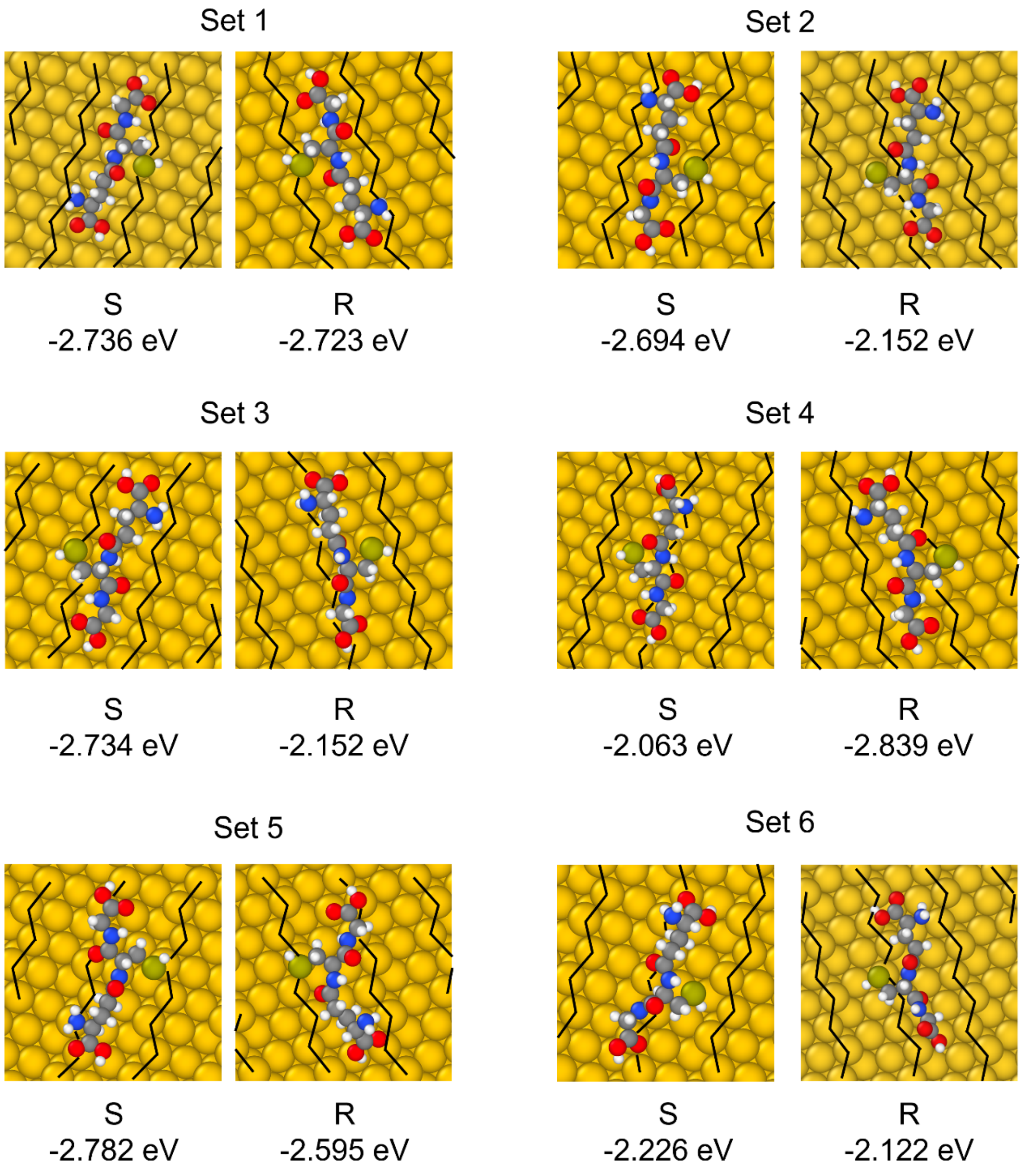

Figure S12: The optimized configurations of six sets of rationally designed initial configurations for L-GSH adsorbed on $Au(321)^{R/S}$. The rationale for initial configuration design is described in the main text.

The configuration of Set 4R shows the strongest binding and is also shown in the main text. The adsorption configurations could be divided into two types: 1) the heteroatoms bond with two steps, such as Sets 1R, 1S, 2S, 3S, 4R, 5S, and 5R, and 2) the backbone interacts with a single step, such as Sets 2R, 3R, 4S, 6R, and 6S. The first type has significantly stronger binding (-2.6~-2.8 eV)

than the second type (-2.0~-2.2 eV), underscoring the importance and high priority of the S and N atoms in the side chain for bonding to the kinked step and supporting our adsorption configuration design rationale. Another piece of evidence for prioritizing the N atom over the O atom can be found in set 5R, where the S atom interacts with one step and an O atom interacts with another step instead of the N atom, leading to weaker adsorption.

Supplementary table for previous DFT work on chiral ligand adsorption

Authors	Molecule(s)	Facet(s)	Package(s) used	Functionals	Key takeaways
Greber et al. [1]	D- and L-Cys	Au(17 11 9)S	DFT with the DACAPO computer program[2]	Repeated slab approaches[3] and ultrasoft pseudopotential approximation[4,5]	D-Cys is ~0.14 eV stronger bingding than L-Cys on Au(17 11 9)S.
Fajin et al. [6]	D- and L-Cys, neutral and zwitterionic state, high and low converage	Au(111), Au(321)R, Au(321)S	DFT with VASP	GGA-PW91[7] and PBE-D2[8] were used for exchange-correlation. PAW method[9,10] was used for core-valence treatment.	Only consider the cases of low coverage with neutral Cys on Au(321). For L-Cys, PW91 shows stronger binding on Au(321)S but PBE-D2 shows stronger binding on Au(321)R. For D-Cys, both PW91 and PBE-D2 shows stronger binding on Au(321)R.
Idris et al. [11]	L-Cys	Au(111) with vacancy and defect	DFT with SIESTA code[12,13]	PBE exchange-correlation functional[14,15]; Tkatchenko–Scheffler van der Waals correction (vdW-TS) [16]; Troullier–Martins norm-conserving pseudopotentials[17]	The coordination number (CN) of the Au binding site makes difference in adsorption strength. Au sites with lower CN tend to bind more strongly. The unconventional zwitterion (H from -SH to -NH2) is the most stable on Au(111) with defect. The conventional Zwitterionic (H from -OH to -NH2) forms are unstable in this work.
Rankin and Sholl [18]	Glycine adlayers	Cu(110)	DFT with VASP	Ultrasoft pseudo-potentials; LDA; GGA. (GGA is more reliable)	Gly is achiral in gas phase, but can become chiral when chemisorbed on atomically flat surfaces (C-H bond angle difference relative to the surface normal). Glycinate molecule is the test monomer. Monomer adsorption and heterochiral/homochiral domains are tested. Heterochiral is preferred.
Barbosa [20]	Bitartrate (R,R-; S,S-; R,S-; S,R-)	Cu(110)	DFT with VASP	LDA parameterized with the Perdew–Zunger exchange–correlation functional; GGA corrections for nonlocality using the Perdew–Wang 1991 (PW91) functional	S,S-bitartrate is 10 kJ/mol less favored in the surface chanel formed by R,R- bitratrate.

Hammer [21]	(S)- and (R)-2-amino-3-(dimethylphosphino)-1-propanethiol (APPT)	Au(17 11 9)S	DACAPO DFT code	GGA (RPBE) exchange-correlation, and periodic boundary conditions; Full relaxation of both molecule and surface atoms	S-enantiomer is more strongly binding to Au(17 11 9)S than R-enantiomer by 8.8 kJ/mol. The preferred adsorption site is the kink site along a monatomic step edge. APPT binds through three functional groups—thiolate (S), phosphino (P), and amino (N)—forming three molecule–surface bonds
Rankin and Sholl [22]	alanine adlayers, enantiopure (all L or all D) versus racemic (L + D mixed)	Cu(110)	DFT with VASP	GGA and ultrasoft pseudopotentials; 7-layer slab (bottom four fixed)	For enantiopure alanine, the adlayer matches the heterochiral glycine motif. The alanine molecules retain the local chirality. For racemic alanine, the adlayer is ordered, but R- and S-alanine are randomly distributed. The energy difference between the most stable enantiopure and racemic adlayers is less than 0.5 kcal/mol/unit cell
Bhatia and Sholl [23]	FAM; PO	Cu(874)S	DFT with VASP	GGA-PW91	R-FAM binds 0.13 eV more strongly than S-FAM on Cu(874)S, which is considered as large enantiospecific adsorption-energy difference. The energy difference mainly comes from the local environment of F: the more stable R enantiomer places F farther from the surface, consistent with fluorine being unfavorable close to Cu. R-PO is only 0.02 eV more stable. The smaller difference is rationalized because both enantiomers interact with the step edge through the same atoms (both H), limiting how different the two adsorption geometries/energies can be.
Rankin and Sholl [24]	Glycine; alanine	Cu(3 1 17)S	DFT with VASP	PW91-GGA; the amino acids adsorb in deprotonated form (glycinate/alaninate)	Experimentally observed adsorbate-induced faceting of Cu(100) into Cu(3,1,17) facets when these amino acids adsorb and the surface is annealed. Both Cu(3,1,17)S and Cu(3,1,17)R facets are observed. Very small enantioselectivity of S- and R-alanine. For racemic alanine on Cu(100) to Cu(3 1 17), there is no strong driving force for spontaneous segregation of enantiomers. However, faceting from Cu(100) to Cu(3 1 17) is thermodynamically favorable for a dense glycine adlayer (not low coverage).

[1] *Phys. Rev. Lett.* **2006**, *96* , 056103

[2] *Phys. Rev. B* **1999**, *59* , 7413.

[3] *Rev. Mod. Phys.* **1992**, *64* , 1045.

[4] *Phys. Rev. B* **1990**, *41* , R7892.

[5] *Phys. Rev. B* **1993**, *47* , 10142.

[6] *Langmuir* **2013**, *29* , 8856–8864
[7] *Phys. Rev. B* **1992**, *46* , 6671-87
[8] *J. Compute. Chem.* **2006**, *27* , 1787-1799
[9] *Phys. Rev. B* **1994**, *50* , 17953-17979
[10] *Phys. Rev. B* **1999**, *59* , 1758-1775
[11] *J. Phys. Chem. C* **2025**,*129* ,13992-14001
[12] *Int. J. Quantum Chem.* **1997**, *65* , 453-461
[13] *J. Condens. Matter Phys.* **2002**, *14* , 2745-2779
[14] *Phys. Rev. Lett.* **1996**, *77* , 3865-3868
[15] *Phys. Rev. Lett.* **1997**, *78* , 1396
[16] *Phys. Rev. Lett.* **2009**, *102* , 073005-073007
[17] *Phys. Rev. B* **1991**, *43* , 1993– 2006
[18] *Surf. Sci.* **2004**, *548* , 301–308
[19] *Curr. Opin. Colloid Interface Sci.* **2008**, *13* , 60–64
[20] *J. Am. Chem. Soc.* **2001**, *123* , 6639–6648
[21] *J. Am. Chem. Soc.* **2002**, *124* , 14789-14794
[22] *Surf. Sci.* **2005**, *574* , L1–8
[23] *Angew. Chemie. Int. Ed.* **2005**, *44* , 7761 –7764
[24] *Langmuir* **2006**, *22* , 8096-8103

References:

[1] BN Dutta and B Dayal. Lattice constants and thermal expansion of gold up to 878° C by X-ray method. *Physica Status Solidi (b)* **1963**, *3*, 473–477.

[2] Wheeler P Davey. Precision measurements of the lattice constants of twelve common metals. *Physical Review* **1925**, *25*, 753.

[3] Eric R Jette and Frank Foote. Precision determination of lattice constants. *The Journal of Chemical Physics* **1935**, *3*, 605–616.

[4] B Höffling et al. Single cysteine adsorption on Au (110): A first-principles study. *Physical Review B* **2010**, *84*, 045407.

[5] Weiwei Ju et al. Au cluster adsorption on perfect and defective MoS 2 monolayers: structural and electronic properties. *Physical Chemistry Chemical Physics* **2017**, *19*, 20735–20748.
